# Cardiac biomarkers and effects of aficamten in obstructive hypertrophic cardiomyopathy: the SEQUOIA-HCM trial

**DOI:** 10.1093/eurheartj/ehae590

**Published:** 2024-09-01

**Authors:** Caroline J Coats, Ahmad Masri, Roberto Barriales-Villa, Theodore P Abraham, Douglas Marshall Brinkley, Brian L Claggett, Albert Hagege, Sheila M Hegde, Carolyn Y Ho, Ian J Kulac, Matthew M Y Lee, Martin S Maron, Iacopo Olivotto, Anjali T Owens, Scott D Solomon, Jacob Tfelt-Hansen, Hugh Watkins, Daniel L Jacoby, Stephen B Heitner, Stuart Kupfer, Fady I Malik, Lisa Meng, Amy Wohltman, James L Januzzi

**Affiliations:** School of Cardiovascular and Metabolic Health, University of Glasgow, Glasgow, UK; Division of Cardiology, Department of Medicine, Oregon Health & Science University, Portland, OR, USA; Department of Cardiology, Complexo Hospitalario Universitario A Coruña, INIBIC, CIBERCV-ISCIII, A Coruña, Spain; UCSF Adult Cardiac Echocardiography Lab, University of California San Francisco, San Francisco, CA, USA; Division of Cardiovascular Medicine, Vanderbilt Heart & Vascular Institute, Nashville, TN, USA; Cardiovascular Division, Brigham and Women’s Hospital, Harvard Medical School, Boston, MA, USA; Département de Cardiologie, Assistance Publique Hôpitaux de Paris, Hôpital Européen Georges-Pompidou, Paris, France; Cardiovascular Division, Brigham and Women’s Hospital, Harvard Medical School, Boston, MA, USA; Department of Medicine, Brigham and Women’s Hospital, Harvard Medical School, Boston, MA, USA; Cardiovascular Division, Brigham and Women’s Hospital, Harvard Medical School, Boston, MA, USA; School of Cardiovascular and Metabolic Health, University of Glasgow, Glasgow, UK; Department of Cardiology, Lahey Hospital and Medical Center, Burlington, MA, USA; Division of Cardiology, Meyer Children’s Hospital, Istituto di Ricovero e Cura a Carattere Scientifico, Florence, Italy; Center for Inherited Cardiovascular Disease, Division of Cardiovascular Medicine, Department of Medicine, University of Pennsylvania Perelman School of Medicine, Philadelphia, PA, USA; Cardiovascular Division, Brigham and Women’s Hospital, Harvard Medical School, Boston, MA, USA; Section of Forensic Genetics, Department of Forensic Medicine, Faculty of Health and Medical Sciences, University of Copenhagen, Copenhagen, Denmark; Department of Cardiology, Copenhagen University Hospital Rigshospitalet, Copenhagen, Denmark; Radcliffe Department of Medicine, University of Oxford, Oxford, UK; Clinical Research, Cytokinetics, Incorporated, South San Francisco, CA, USA; Clinical Research, Cytokinetics, Incorporated, South San Francisco, CA, USA; Clinical Research, Cytokinetics, Incorporated, South San Francisco, CA, USA; Clinical Research, Cytokinetics, Incorporated, South San Francisco, CA, USA; Clinical Research, Cytokinetics, Incorporated, South San Francisco, CA, USA; Clinical Research, Cytokinetics, Incorporated, South San Francisco, CA, USA; Division of Cardiology, Department of Medicine, Massachusetts General Hospital and Harvard Medical School, 55 Fruit Street, Boston, MA 02114, USA; Clinical Trials, Baim Institute for Clinical Research, Boston, MA, USA

**Keywords:** Natriuretic peptides, Troponin, Hypertrophic cardiomyopathy, Aficamten

## Abstract

**Background and Aims:**

The role of biomarker testing in the management of obstructive hypertrophic cardiomyopathy is not well defined. This pre-specified analysis of SEQUOIA-HCM (NCT05186818) sought to define the associations between clinical characteristics and baseline concentrations of N-terminal pro-B-type natriuretic peptide (NT-proBNP) and high-sensitivity cardiac troponin I (hs-cTnI), and to evaluate the effect of treatment with aficamten on biomarker concentrations.

**Methods:**

Cardiac biomarkers were measured at baseline and serially throughout the study. Regression analyses determined predictors of baseline NT-proBNP and hs-cTnI concentrations, and evaluated whether early changes in these biomarkers relate to later changes in left ventricular outflow tract gradient (LVOT-G), other echocardiographic measures, health status, and functional capacity.

**Results:**

Baseline concentration of NT-proBNP was associated with LVOT-G and measures of diastolic function, while hs-cTnI was associated with left ventricular thickness. Within 8 weeks of treatment with aficamten, NT-proBNP was reduced by 79% (95% confidence interval 76%–83%, *P* < .001) and hs-cTnI by 41% (95% confidence interval 32%–49%, *P* < .001); both biomarkers reverted to baseline after washout. Reductions in NT-proBNP and hs-cTnI by 24 weeks were strongly associated with a lowering of LVOT-G, improvement in health status, and increased peak oxygen uptake. N-Terminal pro-B-type natriuretic peptide reduction strongly correlated with the majority of improvements in exercise capacity. Furthermore, the change in NT-proBNP by Week 2 was associated with the 24-week change in key endpoints.

**Conclusions:**

N-Terminal pro-B-type natriuretic peptide and hs-cTnI concentrations are associated with key variables in obstructive hypertrophic cardiomyopathy. Serial measurement of NT-proBNP and hs-cTnI appears to reflect clinical response to aficamten therapy.

## Structured Graphical Abstract

**Structured Graphical Abstract:**
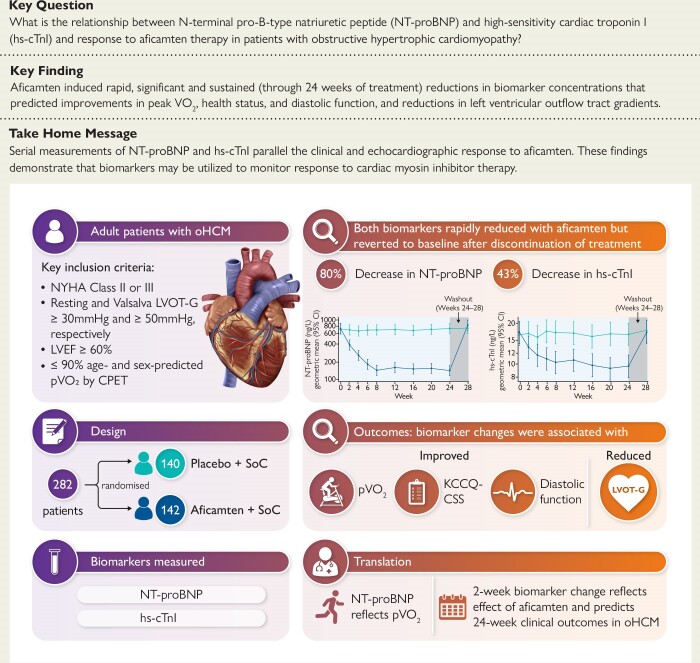
Among individuals treated with aficamten versus placebo in the SEQUOIA HCM trial, concentrations of NT-proBNP and hs-cTnI were significantly associated with cardiac structure and function at baseline. Following treatment with aficamten, NT-proBNP and hs-cTnI concentrations rapidly fell as early as 2 weeks after randomization. Changes in NT-proBNP very strongly predicted impact of aficamten on the primary endpoint of peak VO_2_ change.

**See the editorial comment for this article ‘Natriuretic peptides and cardiac hs-troponins as surrogates of cardiomyocyte stress: clinical value in hypertrophic cardiomyopathy?’, by E. Giannitsis** ***et al*** **., https://doi.org/10.1093/eurheartj/ehae600.**

## Introduction

Hypertrophic cardiomyopathy (HCM) is a chronic myocardial disease characterized by sarcomere hypercontractility.^1^ It manifests clinically as cardiac hypertrophy often accompanied by dynamic left ventricular outflow tract (LVOT) obstruction on cardiac imaging, with arrhythmias and symptoms of dyspnoea, chest pain, and syncope. Abnormal myocardial relaxation is also a prominent feature of HCM and contributes to the symptoms.

Clinical practice guidelines for HCM recommend longitudinal patient follow-up with serial echocardiographic imaging to objectively monitor disease severity and progression.^2,3^ The clinical utility of assessing cardiac biomarkers in HCM has not been firmly established but may provide adjunctive information to clinical and imaging evaluation. In this regard, concentrations of N-terminal pro-B-type natriuretic peptide (NT-proBNP) and high-sensitivity cardiac troponin I (hs-cTnI) are often elevated in HCM, with the degree of elevation associated with adverse outcomes, as well as heart failure symptoms and functional or structural anatomic abnormalities.^4–9^ These biomarkers are inexpensive and readily accessible in most clinical settings. However, due to limited well-controlled prospective data, it is unclear how they could inform clinical practice. More information is needed regarding the potential role for NT-proBNP and hs-cTnI measurement in guiding the care of patients with HCM and, therefore, providing a foundation for clinical practice utility. This may be particularly relevant with the emergence of cardiac myosin inhibitors (CMIs) as a new treatment for HCM.

Cardiac myosin inhibitors are a new treatment class that target the hypercontractility in HCM by reducing myosin binding with actin.^10^ Although there are limited direct data regarding the clinical impact of biomarker dynamics in obstructive HCM (oHCM) specifically, available data suggest that CMIs may be effective at reducing both NT-proBNP and hs-cTnI.^11,12^ However, the significance of this finding remains incompletely explored.

The recent Safety, Efficacy, and Quantitative Understanding of Obstruction Impact of Aficamten in HCM (SEQUOIA-HCM) trial provides an opportunity for the in-depth analysis of the effect of aficamten on biomarker concentrations as well as its clinical associations with biomarker levels. The trial demonstrated that aficamten improved exercise capacity, health status, and symptoms; lowered LVOT gradient (LVOT-G); and reduced guideline indications for septal reduction therapy in oHCM.^13^ Change from baseline of cardiac biomarkers was an exploratory endpoint in the trial. Due to its physicochemical properties, aficamten doses can be adjusted as early as 2 weeks after initiation.^14^ This allows testing of the hypothesis that early change in NT-proBNP and hs-cTnI might inform subsequent efficacy and safety of aficamten. The goals of this pre-specified analysis were therefore to define the associations between clinical characteristics and baseline concentrations of NT-proBNP and hs-cTnI, and to evaluate whether early changes in NT-proBNP and hs-cTnI would relate to later changes in LVOT-G, other echocardiographic measures, health status, and functional capacity.

## Methods

The study was conceived, designed, and conducted by an academic steering committee in conjunction with the study sponsor. Results were generated based on a pre-specified statistical analysis plan (see Supplementary data online, *Statistical Analysis Plan*).^13,15^ The trial was approved by the regulatory agencies in the participating countries and by the institutional review board or independent Ethics Committee at each trial centre. The trial was conducted in accordance with the Declaration of Helsinki, and all study participants provided informed consent prior to participation.

### Study design and patient population

The rationale and design of SEQUOIA-HCM (NCT05186818) has been previously described.^15^ The trial was a phase III, randomized, placebo-controlled, double-blind, multicentre study that enrolled individuals with symptomatic oHCM on stable background medical therapy. Key inclusion criteria were patients aged 18–85 years; New York Heart Association (NYHA) class II or III with echocardiogram core laboratory-determined obstruction (resting and Valsalva LVOT-G ≥30 and ≥50 mmHg, respectively); and left ventricular ejection fraction (LVEF) ≥60% at screening. Participants were required to demonstrate impaired exercise capacity at baseline, defined as ≤90% age- and sex-predicted peak oxygen consumption (pVO_2_) by cardiopulmonary exercise testing (CPET) per core laboratory interpretation. Exclusion criteria included prior treatment with septal reduction therapy (myectomy or alcohol septal ablation); clinically known obstructive coronary artery disease (>70% stenosis in one or more epicardial coronary arteries); history of myocardial infarction; and estimated glomerular filtration rate <30 mL/min/1.73 m^2^. There were no inclusion or exclusion criteria related to either NT-proBNP or hs-cTnI.

### Randomization and intervention

Participants who met the study criteria underwent baseline evaluations, including medical history, physical examinations, vital signs, echocardiography, CPET, laboratory assessments, and health status assessments including Kansas City Cardiomyopathy Questionnaire (KCCQ) measurement. Participants were randomly assigned 1:1, with the use of an automated web-based system, to receive either aficamten or matching placebo for 24 weeks. Participants receiving aficamten were assigned a starting dose of 5 mg, with three opportunities (at Weeks 2, 4, and 6) for dose escalation in 5 mg increments to a maximum dose of 20 mg. Aficamten dose was adjusted to achieve Valsalva LVOT-G <30 mmHg while maintaining LVEF ≥50%. Cardiac biomarkers were measured at baseline, at each dose titration (Weeks 2, 4, and 6), every 4 weeks during treatment, and 4 weeks after drug washout. Participants, investigators, and the sponsor were masked to the echocardiograms and NT-proBNP results throughout the conduct of the study, but not to hs-cTnI for safety reasons.

### Biomarker measurement

Venous blood samples were obtained at each study visit (see Supplementary data online, *Figure S1*), under resting conditions. Biomarkers were measured in a core laboratory in accordance with laboratory manual operating procedures. Plasma NT-proBNP (Elecsys proBNP II, Roche Diagnostics GmbH) was measured on a COBAS 8000 platform; the measuring range for the assay is 10–35 000 ng/L, and the assay had a total percentage (%) imprecision from 2.9% to 5.3% between concentrations of 90–30 668 ng/L. The upper reference limit for this assay in non-acute settings is 125 ng/L. Serum hs-cTnI (Abbott Diagnostics) was measured on an Architect platform; the measuring range for the assay is 3.5–5000 ng/L, and the assay had a total percentage imprecision of 2.5%–9.9% between concentrations of 10.2–2041 ng/L. The upper reference limit for this assay corresponds to the 99th percentile for a healthy reference population and is 14 ng/L for females and 35 ng/L for males.

### Outcome measures in SEQUOIA-HCM

The primary endpoint of the SEQUOIA-HCM trial was change in pVO_2_ over 24 weeks. Key secondary endpoints were change from baseline to Week 24 in KCCQ Clinical Summary Score (KCCQ-CSS), NYHA class, and Valsalva LVOT-G. Exploratory endpoints included changes in cardiac biomarkers and measures of diastolic function (E/e′, left atrial volume). A key safety endpoint was occurrence of core laboratory-measured LVEF <50%.

### Statistical analysis

Normally distributed data were presented as mean ± standard deviation (SD) and categorical data as numbers with percentages (%). N-Terminal pro-B-type natriuretic peptide, hs-cTnI levels, and other non-normally distributed variables are presented as median and interquartile range (IQR). Differences in baseline characteristics between those with biomarker values above vs. below the median were compared using *t*-tests, Wilcoxon rank-sum tests, and *χ*^2^ tests, as appropriate.

Geometric mean and 95% confidence intervals (CIs) for each biomarker are presented at each study visit for each treatment arm. Associations between baseline biomarker values and other baseline covariates were assessed using univariate and multivariable linear regression models with the log-transformed biomarker value as the outcome variable. Multivariable models were selected using backwards stepwise variable selection with a *P*-value threshold of .001. Patients were classified according to quartiles of their relative biomarker changes from baseline to Week 24, and the mean values of pVO_2_ change (Week 24 divided by baseline) were estimated according to these quartiles. Restricted cubic splines with three knots^16^ were used to assess the potentially non-linear associations between log-transformed baseline biomarker values and other baseline variables as well as between log-transformed biomarker changes from baseline and other Week 24 outcomes, adjusted for corresponding baseline values. These associations were estimated both for the overall cohort and separately by treatment arm. Similar linear regression models were fit in order to assess the associations between early biomarker changes (baseline to Weeks 2, 4, 8, and 24, respectively) and 24-week changes in other outcomes, aiming to assess the consistency of these associations across time.

Linear regression models for the outcome of Week 24 pVO_2_ change, both with and without baseline and follow-up biomarker variables, were compared in order to estimate how much of the treatment effect of aficamten was indirectly reflected in NT-proBNP and/or hs-cTnI changes.

All estimates of treatment effects on post-baseline biomarkers included log-transformed baseline value, treatment group, and randomization stratification factors (beta-blocker use and CPET modality) as model covariates.

Two-sided *P* < .05 was considered statistically significant. Week 24 NT-proBNP and hs-cTnI values each had <5% missingness, and no imputations were performed; no study participants died during the trial. Statistical analyses were performed by Brigham and Women's Hospital Clinical Trials Outcomes Center using Stata version 18 (StataCorp, College Station, TX, USA).

## Results

### Patient population

Study schema and CONSORT diagrams are provided as Supplementary data online, *Figure S1*. Between 1 March 2022 and 15 May 2023, eligible individuals were randomized to aficamten or placebo at 101 sites in 14 countries. Of the 282 patients randomized in SEQUOIA-HCM, 277 (98%) had concentrations of NT-proBNP and 270 (96%) had concentrations of hs-cTnI available at baseline. Among these study participants, the median baseline NT-proBNP concentration was 788 ng/L (IQR 346–1699) and hs-cTnI, 12.1 ng/L (IQR 7.7–27.3). The NT-proBNP concentration was >125 ng/L in 254 (92%) patients, and hsTnI was above the reference range in 45 (28%) males and 34 (31%) females. Fewer females than males had NT-proBNP below the median and hs-cTnI above the median.

As shown in *Table 1*, baseline concentrations of NT-proBNP above the median were significantly associated with female sex, prevalent history of paroxysmal atrial arrhythmia, and lower body mass index. Higher NT-proBNP concentrations were also associated with worse performance on baseline CPET (including lower pVO_2_). On echocardiography, an NT-proBNP above the median was significantly associated with higher resting and Valsalva LVOT-G, and with greater left atrial size, and maximal left ventricular (LV) wall thickness but was not associated with LVEF. Higher concentrations of hs-cTnI were significantly associated with male sex, higher KCCQ-CSS, and greater maximal LV wall thickness.

**Table 1:** Baseline characteristics categorized according to median N-terminal pro-B-type natriuretic peptide and high-sensitivity cardiac troponin I

	NT-proBNP			hs-cTnI		
	Below median (*n* = 139)	Above median (*n* = 138)	*P-*value	Below median (*n* = 136)	Above median (*n* = 134)	*P-*value
Randomized to aficamten	66 (47.5)	73 (52.9)	.37	64 (47.1)	75 (56.0)	.14
Age	57 [50, 67]	61 [51, 71]	.032	61 [52, 69]	58 [47, 68]	.10
Female sex	40 (28.8)	72 (52.2)	<.001	68 (50.0)	40 (29.9)	<.001
Race^a^			.51			.09
Asian	27 (19.4)	27 (19.6)		20 (14.7)	33 (24.6)	
Black	1 (0.7)	2 (1.4)		0 (0.0)	2 (1.5)	
Other	2 (1.4)	0 (0.0)		1 (0.7)	1 (0.7)	
White	109 (78.4)	109 (79.0)		115 (84.6)	98 (73.1)	
Geographic region			.59			.015
China	23 (16.5)	23 (16.7)		17 (12.5)	29 (21.6)	
North America	50 (36.0)	42 (30.4)		55 (40.4)	34 (25.4)	
Rest of the world	66 (47.5)	73 (52.9)		64 (47.1)	71 (53.0)	
Medical history						
History of HTN	81 (58.3)	61 (44.2)	.019	75 (55.1)	66 (49.3)	.33
HCM genotype positive	18 (12.9)	30 (21.7)	.05	24 (17.6)	24 (17.9)	.95
Family history of HCM	34 (24.5)	39 (28.3)	.47	35 (25.7)	38 (28.4)	.63
Paroxysmal AF	13 (9.4)	26 (18.8)	.023	20 (14.7)	18 (13.4)	.76
Permanent AF	0 (0.0)	3 (2.2)	.08	0 (0.0)	3 (2.2)	.08
Coronary artery disease	14 (10.1)	20 (14.5)	.26	15 (11.0)	20 (14.9)	.34
Diabetes	13 (9.4)	9 (6.5)	.38	9 (6.6)	12 (9.0)	.47
Vital signs at baseline						
Systolic BP (mmHg)	125 [116, 135]	120 [111, 136]	.33	123 [114, 132]	124 [111, 140]	.39
Diastolic BP (mmHg)	75 [68, 83]	74 [66, 79]	.026	73 [67, 80]	75 [68, 82]	.16
Heart rate (b.p.m.)	70 [62, 80]	66 [60, 74]	.012	68 [61, 78]	67 [61, 76]	.55
BMI (kg/m^2^)	29 [26, 32]	27 [24, 30]	<.001	29 [26, 32]	28 [25, 30]	.040
Background HCM therapy						
Beta-blockers	75 (54.0)	95 (68.8)	.011	90 (66.2)	74 (55.2)	.07
Calcium channel blockers	43 (30.9)	37 (26.8)	.45	33 (24.3)	46 (34.3)	.07
Disopyramide	13 (9.4)	23 (16.7)	.07	21 (15.4)	13 (9.7)	.16
ICD	16 (11.5)	22 (15.9)	.28	16 (11.8)	22 (16.4)	.27
KCCQ-CSS	78 [62, 89]	81 [61, 91]	.59	75 [60, 88]	82 [69, 90]	.008
NYHA class			.54			.25
II	107 (77.0)	102 (73.9)		98 (72.1)	105 (78.4)	
III	32 (23.0)	35 (25.4)		38 (27.9)	28 (20.9)	
IV	0 (0.0)	1 (0.7)		0 (0.0)	1 (0.7)	
Blood biomarkers						
NT-proBNP (ng/L)	346 [219, 521]	1714 [1087, 2709]	<.001	511 [279, 1112]	1064 [542, 2359]	<.001
hs-cTnI (ng/L)	10 [6,19]	16 [10, 38]	<.001	8 [5, 10]	28 [17, 68]	<.001
eGFR (mL/min)	90 [75, 106]	84 [71, 102]	.12	86 [71, 102]	90 [75, 105]	.19
Cardiopulmonary exercise testing						
Total workload (W)	131 [103, 160]	110 [81, 136]	<.001	115 [90, 151]	121 [94, 150]	.21
pVO_2_ (mL/kg/min)	19.6 [16.7, 22.2]	17.1 [14.1, 20.4]	<.001	18.2 [14.5, 20.6]	18.8 [15.3, 21.9]	.11
% predicted pVO_2_	58.9 [49.1, 67.3]	56.0 [47.0, 65.7]	.07	56.0 [49.0, 66.1]	57.9 [47.3, 65.7]	.98
RER	1.16 [1.10, 1.23]	1.17 [1.11, 1.25]	.26	1.18 [1.12, 1.25]	1.15 [1.09, 1.22]	.007
Echocardiogram						
Resting LVOT-G (mmHg)	39 [27, 65]	64 [43, 83]	<.001	50 [30, 72]	52 [32, 74]	.48
Valsalva LVOT-G (mmHg)	74 [57, 95]	90 [70, 109]	<.001	80 [60, 100]	86 [61, 101]	.40
LVEF (%)	76 [72, 79]	75 [71, 78]	.33	76 [72, 78]	74 [70, 78]	.12
Maximal LV wall thickness (cm)	2.01 [1.88, 2.19]	2.08 [1.89, 2.29]	.020	2.00 [1.86, 2.13]	2.12 [1.94, 2.36]	<.001
LAVi (mL/m^2^)	36 [29, 42]	42 [35, 52]	<.001	38 [31, 46]	40 [32, 49]	.20
Septal E/e′	15.8 [12.8, 20.0]	20.8 [16.6, 25.9]	<.001	17.3 [14.3, 23.0]	19.4 [15.0, 23.4]	.14
Lateral E/e′	11.9 [9.5, 16.2]	16.2 [12.5, 21.5]	<.001	13.9 [10.0, 18.2]	14.9 [10.7, 19.4]	.15

Data are shown as *n* (%) or the median [IQR].

AF, atrial fibrillation; BMI, body mass index; BP, blood pressure; eGFR, estimated glomerular filtration rate; HCM, hypertrophic cardiomyopathy; HTN, hypertension; hs-cTnI, high-sensitivity cardiac troponin I; ICD, implantable cardioverter defibrillator; IQR, interquartile range; KCCQ-CSS, Kansas City Cardiomyopathy Questionnaire Clinical Summary Score; LAVi, left atrial volume index; LV, left ventricular; LVEF, left ventricular ejection fraction; LVOT-G, left ventricular outflow tract gradient; NT-proBNP, N-terminal pro-B-type natriuretic peptide; NYHA, New York Heart Association; pVO_2_, peak oxygen uptake; RER, respiratory exchange ratio.

^a^Race was denoted by the patient.

Cubic spline graphs with univariate correlation analyses showed significant associations between baseline biomarker levels and key echocardiographic characteristics of oHCM (see Supplementary data online, *Figures S2* and *S3*). Multivariate linear regression analyses identified predictors of baseline biomarker values (*Tables 2* and *3*). Independent predictors of baseline NT-proBNP concentrations included concentrations of hs-cTnI; measurements reflecting diastolic dysfunction [left atrial volume index (LAVi); the ratio of early transmitral Doppler velocity/septal peak mitral annular velocity during early filling (E/e′)]; body mass index; LVOT-G at rest; and female sex. In contrast to NT-proBNP, independent predictors of hs-cTnI included NT-proBNP concentrations, male sex, and maximal LV wall thickness. Excluding each biomarker led to subtle changes in the predictors of the two; for example, removing NT-proBNP from the model resulted in only maximal LV wall thickness as a predictor of hs-cTnI (see Supplementary data online, *Tables S1* and *S2*).

**Table 2:** Results of multivariable linear regression for predictors of baseline N-terminal pro-B-type natriuretic peptide concentration

Covariate	Univariate			Multivariable		
	Association (95% CI)	|*Z*|	*P*-value	Association (95% CI)	|*Z*|	*P*-value
hs-cTnI (per log)	+35% (+21%, +50%)	5.5	<.001	+35% (+23%, +47%)	6.7	<.001
LAVi (per SD)	+50% (+32%, +70%)	6.3	<.001	+30% (+17%, +45%)	4.9	<.001
E/e′ septal (per SD)	+69% (+50%, +91%)	8.7	<.001	+31% (+17%, +47%)	4.6	<.001
BMI (per 5 kg/m^2^)	−36% (−46%, −23%)	5.0	<.001	−24% (−34%, −13%)	3.9	<.001
Resting LVOT-G (per SD)	+49% (+31%, +70%)	6.2	<.001	+23% (+10%, +37%)	3.7	<.001
Female sex	+58% (+20%, +107%)	3.3	.001	+48% (+18%, +86%)	3.4	.001
E/e′ lateral (per SD)	+55% (+37%, +76%)	6.9	<.001			
LVMi (per SD)	+43% (+25%, +62%)	5.4	<.001			
Valsalva LVOT-G (per SD)	+36% (+20%, +55%)	4.7	<.001			
Max LV wall thickness (per SD)	+33% (+17%, +52%)	4.3	<.001			
History of AF	+57% (+7%, +129%)	2.3	.021			
LVEF (per SD)	−9% (−20%, +4%)	1.4	.18			
LVEDVi (per SD)	−8% (−20%, +5%)	1.3	.21			
NYHA class	+21% (−11%, +65%)	1.2	.22			
Age (per 10 years)	+5% (−6%, +16%)	0.8	.40			
Creatinine (per SD)	−1% (−13%, +14%)	0.1	.93			

AF, atrial fibrillation; BMI, body mass index; CI, confidence interval; E/e′, ratio of early diastolic mitral inflow velocity to early diastolic mitral annulus velocity; hs-cTnI, high-sensitivity cardiac troponin I; LAVi, left atrial volume index; LV, left ventricular; LVEDVi, left ventricular end-diastolic volume index; LVEF, left ventricular ejection fraction; LVOT-G, left ventricular outflow tract gradient; LVMi, left ventricular mass index; NT-proBNP, N-terminal pro-B-type natriuretic peptide; NYHA, New York Heart Association; SD, standard deviation.

**Table 3:** Results of multivariable linear regression for predictors of baseline high-sensitivity cardiac troponin I concentration

Covariate	Univariate			Multivariable		
	Association (95% CI)	|*Z*|	*P*-value	Association (95% CI)	|*Z*|	*P*-value
NT-proBNP (per log)	+41% (+25%, +60%)	5.5	<.001	+41% (+25%, +59%)	5.6	<.001
Female sex	−42% (−57%, −22%)	3.7	<.001	−48% (−61%, −32%)	4.7	<.001
Max LV wall thickness (per SD)	+54% (+35%, +76%)	6.3	<.001	+33% (+17%, +52%)	4.3	<.001
LVMi (per SD)	+45% (+27%, +67%)	5.3	<.001			
Age (per 10 years)	−18% (−26%, −8%)	3.4	.001			
LVEDVi (per SD)	+17% (+1%, +35%)	2.1	.035			
LVEF (per SD)	−13% (−25%, +0%)	1.9	.06			
BMI (per 5 kg/m^2^)	−16% (−31%, +2%)	1.8	.08			
NYHA class	−12% (−37%, +22%)	0.8	.44			
Creatinine (per SD)	+6% (−8%, +22%)	0.8	.45			
LAVi (per SD)	+6% (−9%, +22%)	0.8	.45			
E/e′ lateral (per SD)	+6% (−9%, +23%)	0.7	.46			
Valsalva LVOT-G (per SD)	+5% (−9%, +22%)	0.7	.47			
E/e′ septal (per SD)	+4% (−10%, +21%)	0.6	.56			
Resting LVOT-G (per SD)	+4% (−10%, +21%)	0.6	.56			
History of AF	−8% (−39%, +38%)	0.4	.68			

AF, atrial fibrillation; BMI, body mass index; CI, confidence interval; E/e′, ratio of early diastolic mitral inflow velocity to early diastolic mitral annulus velocity; hs-cTnI, high-sensitivity cardiac troponin I; LAVi, left atrial volume index; LV, left ventricular; LVEDVi, left ventricular end-diastolic volume index; LVEF, left ventricular ejection fraction; LVOT-G, left ventricular outflow tract gradient; LVMi, left ventricular mass index; NT-proBNP, N-terminal pro-B-type natriuretic peptide; NYHA, New York Heart Association; SD, standard deviation.

### Treatment effect of aficamten according to baseline biomarker concentration

The effect of aficamten on the change in pVO_2_ was similar in people with baseline NT-proBNP or hs-cTnI above or below the median (*P* interaction .68 and .22, respectively; Supplementary data online, *Figure S4*).

### Impact of treatment on cardiac biomarker concentration

**Figure 1:**
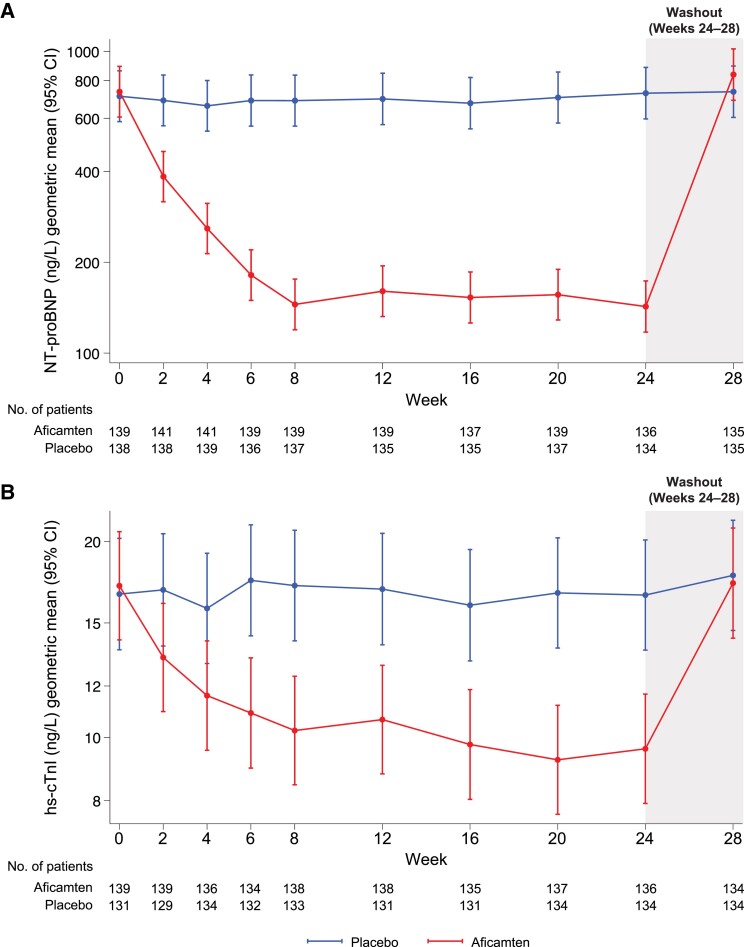
Effect of aficamten on cardiac biomarkers, (*A*) N-terminal pro-B-type natriuretic peptide and (*B*) high-sensitivity cardiac troponin I, over time in SEQUOIA-HCM. After treatment with aficamten, the majority of reduction in N-terminal pro-B-type natriuretic peptide and high-sensitivity cardiac troponin I was seen by Week 8. Treatment was discontinued at Week 24

Aficamten treatment led to an immediate and significant reduction in both NT-proBNP and hs-cTnI concentrations, with substantial lowering of both biomarkers by Week 2 (*Figure 1*). Notably, by Week 8, the lowering of both biomarkers from aficamten treatment was nearly as large as later in the study; NT-proBNP was reduced by 79% (95% CI 76%–83%, *P* < .001) and hs-cTnI by 41% (95% CI 32%–49%, *P* < .001). Biomarker lowering persisted to Week 24 when the relative reduction from baseline was 80% (95% CI 77%–83%, *P* < .001) in NT-proBNP and 43% (95% CI 36%–49%, *P* < .001) in hs-cTnI. Following completion of the treatment period, cessation of aficamten resulted in concentrations of both NT-proBNP and hs-cTnI returning to baseline values.

### Biomarker change and effect of aficamten by 24 weeks

**Figure 2:**
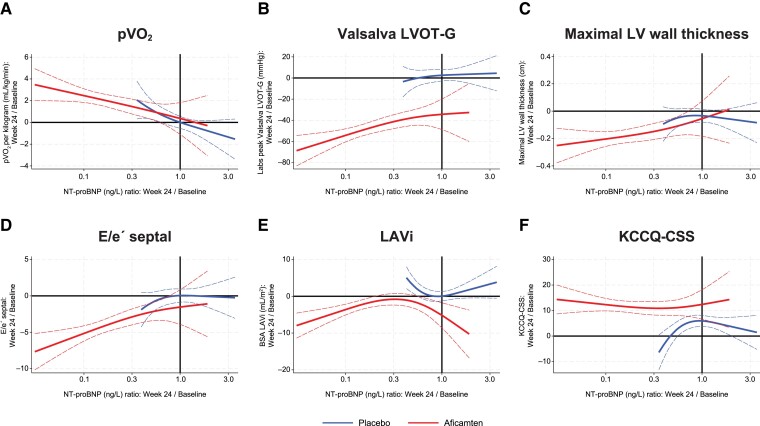
Cubic spline analyses relating changes in N-terminal pro-B-type natriuretic peptide concentration to changes in other clinical measures at 24 weeks in all patients. Linear regression with cubic terms. Analyses were adjusted for baseline values of both parameters. Solid and dotted lines show the association correlate with 95% confidence intervals. Black lines indicate no change from baseline. Significant correlations were found between N-terminal pro-B-type natriuretic peptide at Week 24 and key clinical measures of (*A*) peak oxygen uptake, (*B*) Valsalva left ventricular outflow tract gradient, (*C*) maximal wall thickness, (*D*) E/e′ septal, (*E*) left atrial volume index, and (*F*) Kansas City Cardiomyopathy Questionnaire Clinical Summary Score at the same time point

**Figure 3:**
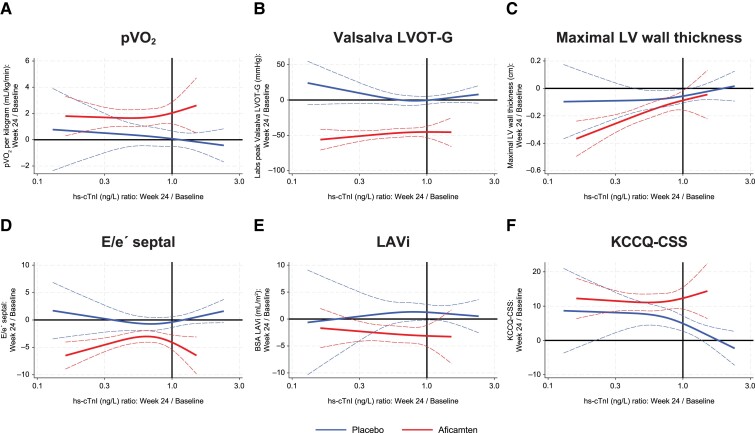
Cubic spline analyses relating changes in high-sensitivity cardiac troponin I concentration to changes in other clinical measures at 24 weeks in all patients. Linear regression with cubic terms. Analyses were adjusted for baseline values of both parameters. Solid and dotted lines show the association correlate with 95% confidence intervals. Black lines indicate no change from baseline. Significant correlations were found between high-sensitivity cardiac troponin I at Week 24 and key clinical measures of (*A*) peak oxygen uptake, (*B*) Valsalva left ventricular outflow tract gradient, (*C*) maximal wall thickness, (*D*) E/e′ septal, (*E*) left atrial volume index, and (*F*) Kansas City Cardiomyopathy Questionnaire Clinical Summary Score at the same time point

The association between change in NT-proBNP and hs-cTnI by Week 24 and change in key clinical measures of oHCM at the same time point are shown in *Figures 2* and *3*. Both biomarkers were found to be inversely correlated with change in pVO_2_ and health status (with increase in KCCQ-CSS) and directly correlated with change in Valsalva LVOT-G, maximal LV wall thickness, and E/e′. N-Terminal pro-B-type natriuretic peptide change by Week 24 was directly correlated with change in LAVi, while hs-cTnI was not.

**Figure 4:**
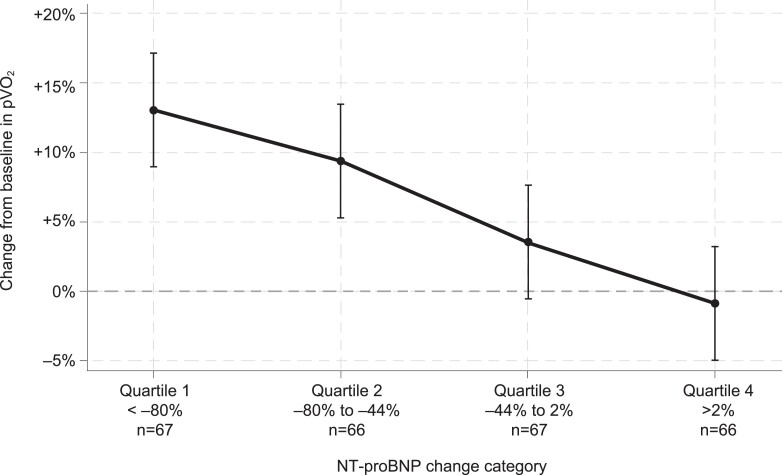
Change in peak oxygen uptake across N-terminal pro-B-type natriuretic peptide change categories. An inverse linear association between relative change in N-terminal pro-B-type natriuretic peptide and change in peak oxygen uptake was observed for all patients. Geometric mean and 95% confidence intervals are shown. Horizontal dashed line indicates no change from baseline

The observed treatment effect of aficamten on Week 24 pVO_2_ change of +1.7 (+1.0, +2.4) mL/kg/min was attenuated to −0.0 (−1.1, +1.1) mL/kg/min after accounting for the concomitant change in NT-proBNP. In contrast, accounting for 24-week hs-cTnI changes had no impact on the estimated treatment effect [+1.7 (+0.8, +2.5) mL/kg/min]. A visual representation of this strong inverse linear association between change in NT-proBNP and change in pVO_2_ is detailed in *Figure 4*. In analyses that combine NT-proBNP and hs-cTnI change relative to change in functional capacity after treatment with aficamten, most of the improvement in pVO_2_ appeared to be associated with NT-proBNP changes (*Table 4*). To better understand if the change in NT-proBNP was driven by improvement in LVOT-G, an analysis of the relationship between the change in NT-proBNP and change in Valsalva LVOT-G over 24 weeks is shown in Supplementary data online, *Figure S5*.

**Table 4:** Change in biomarkers and percentage change in peak oxygen uptake

Mean pVO_2_ change from baseline (%)	Quartile of hs-cTnI change			
Quartile of NT-proBNP change	1 (upper)	2	3	4 (lower)
1 (upper)	+13% (+7%, +19%) [*N* = 36]	+14% (+7%, +22%) [*N* = 18]	+13% (+1%, +24%) [*N* = 9]	+9% (−10%, +28%) [*N* = 3]
2	+10% (+1%, +18%) [*N* = 14]	+4% (−3%, +12%) [*N* = 21]	+15% (+8%, +23%) [*N* = 20]	+1% (−14%, +15%) [*N* = 6]
3	+3% (−8%, +14%) [*N* = 9]	+15% (+5%, +25%) [*N* = 11]	0% (−7%, +8%) [*N* = 23]	+2% (−5%, +10%) [*N* = 21]
4 (lowest quartile)	−2% (−18%, +15%) [*N* = 4]	0% (−11%, +11%) [*N* = 9]	0% (−9%, +10%) [*N* = 12]	0% (−6%, +6%) [*N* = 34]

The study cohort was classified according to quartiles of their relative biomarker changes from baseline to Week 24. This demonstrates that most of the improvement in pVO_2_ was associated with NT-proBNP change.

hs-cTnI, high-sensitivity cardiac troponin I; N number; NT-proBNP, N-terminal pro-B-type natriuretic peptide; pVO_2_, peak oxygen uptake.

### Early change in biomarkers and subsequent effect of aficamten

**Figure 5:**
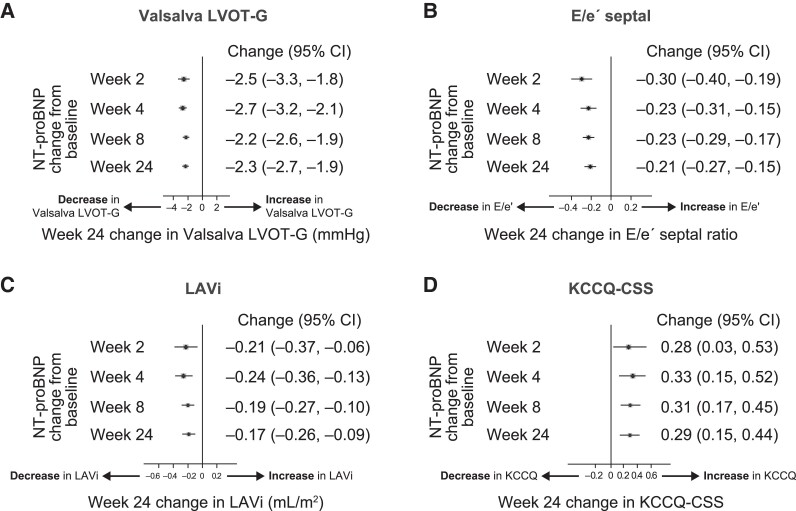
Forest plots of study outcomes at 24 weeks including (*A*) Valsalva left ventricular outflow tract gradient, (*B*) E/e′ septal, (*C*) left atrial volume index, and (*D*) Kansas City Cardiomyopathy Questionnaire Clinical Summary Score according to N-terminal pro-B-type natriuretic peptide measurement at Weeks 2, 4, 8, and 24. Reduction in N-terminal pro-B-type natriuretic peptide as early as 2 weeks after study initiation was associated with later improvement in key clinical measures at Week 24 that were comparable to later N-terminal pro-B-type natriuretic peptide results. Results are expressed per 10% reduction in N-terminal pro-B-type natriuretic peptide. Solid black vertical line indicates no treatment effect

Given the very early change in NT-proBNP and hs-cTnI, we examined the correlation between Week 2 change in these biomarkers and Week 24 change in pVO_2_, Valsalva LVOT-G, maximal LV wall thickness, E/e′, LAVi, and KCCQ-CSS (see Supplementary data online, *Figures S6* and *S7*). By Week 2 of aficamten treatment, the correlations between change in biomarkers and outcome measures were substantially associated with those at Week 24. Furthermore, as shown in *Figure 5*, very early change in NT-proBNP was consistently associated with the later magnitude of proportional change across multiple study endpoints. For example, each 10% reduction in NT-proBNP at Week 2 was associated with a −2.5 mmHg (95% CI −3.3 to −1.8) reduction in Valsalva LVOT-G at Week 24; similar associations existed between Week 2 change in NT-proBNP and later E/e′, LAVi, and KCCQ-CSS. Results of NT-proBNP testing during (Week 4) or at the end (Week 8) of titration were similarly associated with Week 24 outcomes.

### Relationship between left ventricular ejection fraction and N-terminal pro-B-type natriuretic peptide during treatment

Aficamten treatment (using a dose titration protocol) resulted in a modest decrease in LVEF at Week 24 compared with placebo [least squares mean difference −5% (95% CI −6 to −3)] in parallel with significant decreases in NT-proBNP and hs-cTnI (see Supplementary data online, *Figure S8*). A transient reduction in LVEF <50% occurred in 5 (3.5%) study participants in the aficamten group and 1 (0.7%) in the placebo group. Baseline NT-proBNP (*P* = .52) and hs-cTnI (*P* = .37) did not predict future LVEF <50%. An increase in NT-proBNP (+111 ng/L) from baseline was observed in only one study participant with LVEF <50% who was diagnosed with concurrent coronavirus disease 2019 infection. The five other study participants had a decline in NT-proBNP from baseline ranging from −291 to −921 ng/L despite the occurrence of LVEF <50%. One study participant treated with placebo experienced an LVEF <50% with concurrent symptoms and signs of heart failure; their NT-proBNP ranged between 465 and 936 ng/L during the study. No aficamten-treated participant with LVEF <50% had clinical heart failure.

### Individual patient data

Baseline and Week 24 data for biomarkers, LVEF, pVO_2_, and LVOT-G are included in Supplementary data online, *Figure S9*.

## Discussion

In SEQUOIA-HCM, baseline concentrations of NT-proBNP and hs-cTnI were linked to key features that are known to influence exercise intolerance and prognosis in oHCM including LVOT-G, left atrial size, cardiac filling pressures, and LV hypertrophy. Our analyses showed that study participants treated with aficamten achieved rapid and significant improvements in NT-proBNP and hs-cTnI concentrations, which returned to baseline levels after treatment washout, confirming a reversible pharmacodynamic effect. Changes in NT-proBNP and hs-cTnI concentration after 24 weeks of treatment were associated with improvements in cardiac structure and function extending beyond reduction in LVOT-G. Importantly, change in NT-proBNP strongly reflected the impact of aficamten on improved exercise capacity. In *post hoc* analyses, we were also able to show that early change in NT-proBNP (as early as Week 2) was strongly associated with improvements in key measures such as pVO_2_, LVOT-G, LAVi, E/e′, and KCCQ-CSS; accordingly, among those treated with aficamten, early reduction in NT-proBNP might be considered a useful signal of future treatment effect (*Structured Graphical Abstract*). Although improvements in cardiac biomarkers have been observed previously with CMI therapy, this is the first analysis of how the velocity and magnitude of biomarker change relate to clinical outcomes in this setting.

The baseline analysis of biomarker concentrations in SEQUOIA-HCM provides an important understanding of the clinical factors that explain concentrations of NT-proBNP and hs-cTnI among individuals with HCM. Study participants in SEQUOIA-HCM had a wide range of NT-proBNP and hs-cTnI concentrations at baseline, and these biomarkers reflected different aspects of cardiac structure, function, and prognosis in oHCM. For NT-proBNP, the strongest predictors of the biomarker included LVOT-G and echocardiographic measures of diastolic function, two key predictors of symptoms in oHCM. For hs-cTnI, the main cardiac predictor was maximal LV wall thickness. In addition, both biomarkers were independently predictive of each other; presumably, this reflects the inter-relationship between wall stress (NT-proBNP) and troponin release, the latter of which may reflect multiple mechanisms including cardiomyocyte injury and cell death as well as exosomal release under cellular stress.^17^ Prior studies examining associations between NT-proBNP and hs-cTnI relative to myocardial structure and function and response to treatment in oHCM generally support these findings.^18–21^ Notably, female sex has been repeatedly associated with modest but significant effects on biomarker concentrations in other studies, with higher NT-proBNP and lower hs-cTnI compared to males.^22^ We demonstrate the same finding in the present analysis. When measuring either biomarker in a person with HCM, clinicians should be aware of how numerous variables including sex may modestly affect biomarker values; whether sex-based biomarker cut-points are needed for clinical application is uncertain and requires further evaluation.

In SEQUOIA-HCM, treatment with aficamten resulted in a substantial reduction in NT-proBNP and hs-cTnI by 2 weeks, a time when study participants were receiving the lowest dose of the drug and LVOT-G lowering was not yet as large as it was later in the trial. By 8 weeks, the reductions in NT-proBNP and hs-cTnI were nearly as large as achieved later in the trial and were sustained through 24 weeks. Overall, the magnitude of reduction in biomarker concentrations from treatment with aficamten is generally comparable to that reported from treatment with mavacamten.^21^ In this analysis, we demonstrate two important findings: first, the reductions in biomarkers by Week 24 were strongly associated with improvement in key outcomes in oHCM including improved pVO_2_, reduction in LVOT-G, and better health status. Second, the change in NT-proBNP by 24 weeks might be an accurate surrogate for change in pVO_2_. These results suggest that clinicians may utilize NT-proBNP to monitor functional and qualitative response to aficamten, particularly since CPET may not be widely available and requires specialized performance and interpretation. *Post hoc* analyses also showed that early change in NT-proBNP (as early as Week 2) was strongly associated with improvements in key measures such as pVO_2_, LVOT-G, LAVi, E/e′, and KCCQ-CSS; accordingly, among those treated with aficamten, early reduction in NT-proBNP might be considered a useful signal of future treatment effect. More data are needed to understand how biomarkers may be used to monitor efficacy of CMI therapy.

In advanced stage (non-CMI treated) HCM, and other causes of chronic heart failure, a deteriorating LVEF is associated with a rise in concentrations of both NT-proBNP and hs-cTnI.^8,23,24^ Results from SEQUOIA-HCM suggest that in the small number of patients who had an LVEF <50%, a reduction (rather than an increase) was generally seen in NT-proBNP and hs-cTnI. The reasons for the lack of a risk signal in biomarker concentrations remain uncertain. Since transmural myocardial wall stress is a prime driver of NT-proBNP and hs-cTnI release, it is reasonable to hypothesize that aficamten reduced wall stress by mitigating hypercontractility, even in the setting of lower LVEF. Regardless, these data indicate that while NT-proBNP change strongly associates with efficacy measures during aficamten treatment, results from this biomarker cannot replace echocardiography to detect small excursions in LVEF <50%. More real-world data are required to understand if NT-proBNP may have a role in detecting asymptomatic LVEF <40% or in emergency settings where timely access to echocardiography might be limited.

In SEQUOIA-HCM, the decrease in NT-proBNP concentrations following aficamten treatment corresponded with improvement in several structural and functional correlates of HCM, but this study cannot answer which of these variables explains the reductions. When measuring NT-proBNP and hs-cTnI, the change in pVO_2_ appeared best predicted by the former; future studies should examine the meaning of change in hs-cTnI relative to other key measures in oHCM such as cardiac remodelling parameters. As aficamten also reduces NT-proBNP in non-obstructive HCM,^25^ the NT-proBNP decrease documented in this analysis may not be directly related to reduced LVOT-G itself. Rather, it might reflect the effect of aficamten to reduce myocardial hypercontractility, which in turn results in rapid and sustained lowering of LVOT-G and improvement in diastolic abnormalities throughout the treatment period; ultimately, this could hypothetically explain the strong correlation with impact on pVO_2_. Indeed, while the change in pVO_2_ was quite linear with the change in NT-proBNP, the change in LVOT-G was not as clearly seen when considering change in LVOT-G categories. In contrast to NT-proBNP, hs-cTnI was not associated with LVOT-G, diastolic abnormalities, or pVO_2_, but hs-cTnI did change in response to aficamten treatment. Lowering of both biomarkers has also been observed in non-obstructive HCM.^25,26^ We hypothesize that the reduction in hs-cTnI reflects reduced cardiomyocyte stress and injury consequent to improvement in LV wall stress; this reduction in wall stress is identified by reduction in NT-proBNP. More data are needed in this regard.

This study has several limitations. First, a relatively small population was treated for only 24 weeks. Longer-term studies on larger cohorts are needed to fully characterize the impact of CMIs on cardiac biomarkers. Although the association between change in NT-proBNP and pVO_2_ is extremely strong, causality cannot be determined with the design of this study. Nonetheless, the totality of evidence in the analysis supports the interpretation of an association between NT-proBNP change and improvement in functional capacity following aficamten treatment. Lastly, the study participants in SEQUOIA-HCM were mostly White or Asian and few had chronic atrial fibrillation. More data are needed to better understand the applicability of biomarker monitoring to CMI therapy in more diverse populations.

In conclusion, aficamten treatment is associated with a rapid and reversible decrease in cardiac biomarkers. These findings offer mechanistic and clinical insights into the beneficial therapeutic effects of aficamten in oHCM.

## Supplementary Material

ehae590_Supplementary_Data

## Declarations

### Data Availability

Qualified researchers may submit a request containing the research objectives, endpoints/outcomes of interest, statistical analysis plan, data requirements, publication plan, and qualifications of the researcher(s). Requests are reviewed by a committee of internal and external advisers. If approved, data necessary to address the research question will be provided under the terms of the data sharing agreement. Requests will be considered after applications for marketing authorization in the USA and Europe have been reviewed and final decisions rendered. Requests may be submitted to medicalaffairs@cytokinetics.com.

### Pre-registered Clinical Trial Number

SEQUOIA-HCM (NCT05186818).

## Appendix

SEQUOIA-HCM Investigators: Yuhui Zhang (Fuwai Hospital, CAMS & PUMC, China), Haibo Yang (The First Affiliated Hospital of Zhengzhou University, China), Chunli Shao (Peking University Third Hospital, China), Zuyi Yuan (The First Affiliated Hospital of Xi’an Jiaotong University, China), Qingchun Zeng (Nanfang Hospital, China), Xiaodong Li (Shengjing Hospital of China Medical University, China), Changsheng Ma (Beijing Anzhen Hospital, China), Yushi Wang (The First Hospital of Jinlin University, China), Yan Shu (Sichuan Provincial People's Hospital, China), Mulei Chen (Beijing Chao-yang Hospital, Capital Medical University, China), Ling Tao (The First Affiliated Hospital of the Air Force Medical University, China), Xinli Li (Jiangsu Province Hospital, China), Jingfeng Wang (Sun Yat-Sen Memorial Hospital, China), Zaixin Yu (Xiangya Hospital of Central South University, China), Xiang Cheng (Union Hospital affiliated to Tongji Medical College of Huazhong University of Science and Technology, China), Kui Hong (The Second Affiliated Hospital of Nanchang University, China), David Zemanek (Interni klinika kardiologie a angiologie 1. Lekarske fakulty a Vseobecne fakultni nemocnice v Praze, Czech Republic), Henning Bundgaard [Hjertecentret (The Heart Center), Copenhagen University Hospital/Rigshosptalet, Denmark], Jens Thune (Bispebjerg Hospital, University of Copenhagen, Denmark), Morten Jensen (Aarhus Universitetshospital, Denmark), Jens Mogensen (Aalborg University Hospital, Aalborg Sygehus, Denmark), Albert Hagege [Groupement Hospitalier Universitaire Ouest—Hopital Europeen Georges-Pompidou (HEGP), France], Gilbert Habib [Assistance Publique Hopitaux de Marseille (AP-HM)—Hopital de La Timone, France], Philippe Charron (CHU Paris-GH La Pitie Salpetriere-Charles Foix—Hopital Pitie-Salpetriere, France), Thibault Lhermusier (Centre Hospitalier Universitaire de Toulouse—Hopital Rangueil, France), Jean-Noël Trochu (CHU de Nantes—Hopital Nord Laennec, France), Patricia Reant [Centre Hospitalier Universitaire (CHU) du Haut Leveque, France], Damien Logeart (Hopital Lariboisiere, Hospitalier Universitaire Nord, France), Veselin Mitrovic (Kerckhoff-Klinik- Bad Nauheim, Germany), Tarek Bekfani (Universitatsklinikum Magdeburg, Germany), Frank Edelmann (Charite Universitatsmedizin Berlin, Germany), Tim Seidler (Klinikum der Georg-August-Universitaet Goettingen, Germany), Benjamin Meder (Universitatsklinikum Heidelberg, Germany), Paul Christian Schulze (Universitaetsklinikum Jena, Germany), Stephan Stoerk [University of Wurzburg, Comprehensive Heart Failure Center (CHFC), Germany], Tienush Rassaf (Universitaetsklinikum Essen, Germany), Bela Merkely (Semmelweis Egyetem, Varosmajori Sziv es Ergyogyaszati Klinika, Hungary), Donna Zfat-Zwas (Hadassah Medical Center, Israel), Michael Arad (The Chaim Sheba Medical Center, Israel), Majdi Halabi (Ziv MC, Israel), Offir Paz (Kaplan Medical Center, Israel), Xavier Piltz (The Barzilai Medical Center, Faculty of Health Sciences, Ben Gurion University of The Negev, Israel), Iacopo Olivotto/Mattia Targetti (Careggi University Hospital, Italy), Marco Metra (Azienda Ospedaliera Spedali Civili di Brescia-University of Brescia, Italy), Marco Canepa (Ospedale Policlinico San Martino—IRCCS, Italy), Beatrice Musumeci (Azienda Ospedaliera S’Andrea di Roma—UOC Cardiologia, Italy), Michele Emdin (Fondazione Toscana Gabriele Monasterio Per La Ricerca Medica E Di Sanita Pubblica Ftgm, Italy), Michelle Michels (Erasmus Medisch Centrum 1, The Netherlands), Ahmad Amin [Amsterdam University Medical Center (Amsterdam UMC), Academic Medical Center (AMC), The Netherlands], Christian Knackstedt [Maastricht University Medical Center (MUMC), The Netherlands], Artur Oreziak (Narodowy Instytut Kardiologii Stefana kard. Wyszynskiego Panstwowy Instytut Badawczy, Poland), Wojciech Wojakowski (Kardio Brynow Sp. z o.o., Poland), Dariusz Dudek (Krakowskie Centrum Diagnostyczno-Kliniczne, Poland), Alexandra Toste (Hospital Da Luz, Portugal), José Mesquita Bastos (CH Baixo Vouga—Aveiro, Portugal), Roberto Barriales-Villa (Complejo Hospital Universitario A Coruna, Spain), Pablo Garcia Pavia (Hospital Universitario Puerta de Hierro de Majadahonda, Spain), Juan Ramón Gimeno Blanes (Hospital Universitario Virgen de la Arrixaca, Spain), Rafael Jesus Hidalgo Urbano (Hospital Universitario Virgen Macarena-merge, Spain), Ana Garcia Alvarez (Hospital Clinic i Provincial de Barcelona, Spain), Luis Miguel Rincón Diaz (Hospital Universitario de Salamanca, Spain), Tomas Vicente Ripoll Vera (Hospital Son Llatzer, Spain), Perry Elliott (St Bartholomew’s Hospital-Barts Health NHS Trust, UK), Caroline Coats (Gartnaval General Hospital, NHS Greater Glasgow and Clyde, UK), Rob Cooper (Liverpool Heart and Chest Hospital—Liverpool Heart and Chest Hospital NHS Foundation Trust, UK), Masliza Mahmod (John Radcliffe Hospital—Oxford University Hospitals NHS Foundation Trust, UK), William Bradlow (Queen Elizabeth Hospital Birmingham-University Hospitals Birmingham NHS Foundation Trust, UK), Antonis Pantazis (Harefield Hospital—Royal Brompton And Harefield NHS Foundation Trust, UK), Maria Teresa Tome Esteban (St George’s Hospital—St George’s University Hospitals NHS Foundation Trust, UK), Ahmad Masri (Oregon Health and Science University, USA), Michael Nassif (Saint Luke’s Hospital of Kansas City, USA), Ali Marian (Baylor St. Luke’s Medical Center, USA), David Owens (University of Washington Medical Center, USA), Matthew Wheeler (Stanford University, USA), Frank McGrew (Baptist Memorial Health Care, USA), Richard Bach (Washington University School of Medicine, USA), Omar Wever-Pinzon (The University of Utah Health Sciences Center, USA), Elias Collado (Holy Cross Hospital, Fort Lauderdale, USA), Aslan Turer (The University of Texas Southwestern Medical Center, USA), Bashar Hannawi (Henry Ford Medical Center, USA), Jeffrey Geske (Mayo Clinic, USA), Anjali Owens (Penn Heart and Vascular Center—Heart Failure, USA), John Symanski (Sanger Heart & Vascular Institute, Atrium Healthcare, USA), Christopher Kramer (University of Virginia Health System, USA), Nitasha Sarswat (The University of Chicago Medicine, USA), Ferhaan Ahmad (The University of Iowa Hospitals & Clinics, USA), Theodore Abraham (University of California, San Francisco Medical Center, USA), Jeremy Markowitz (University of Minnesota, USA), Neal Lakdawala [Harvard Medical School—Brigham and Women’s Hospital (BWH)—The Schuster Family Transplantation Research Center (TRC), USA], Lubna Choudhury (Northwestern Memorial Hospital, USA), Sandeep Jani (MedStar Franklin Square Medical Center, USA), Marshall Brinkley [Vanderbilt University Medical Center (VUMC)—Vanderbilt University Hospital (VUH), USA], Ozlem Bilen (Emory University Hospital, The Emory Clinic, USA), Craig Asher (Cleveland Clinic Florida, USA), Sitaramesh Emani (Ohio State University Medical Center, USA), Abhinav Sharma (Heart and Vascular Center—Center for Advanced Care—Froedtert Hospital, USA), David Fermin [Spectrum Health Medical Group (SHMG)—Cardiology (West Michigan Heart P.C)—Grand Rapids, USA], Melissa Lyle (Mayo Clinic, USA), David Raymer (UCHealth Heart and Vascular Center—Anschutz Medical Campus, USA), Andrew Darlington (Piedmont Fayette Hospital, USA), Martin Maron (Lahey Hospital & Medical Center, USA), Christopher Nielsen (Medical University of South Carolina, USA), Andrew Wang (Duke University, USA), Sherif Nagueh (Houston Methodist Hospital, USA), Matthew Martinez (Morristown Medical Center, USA), Milind Desai (Cleveland Clinic—Taussig Cancer Institute, USA), Albree Tower-Rader [Massachusetts General Hospital (MGH)—Interventional Cardiology Associates, USA], Jacob Kelly (Alaska Heart and Vascular Institute, USA), Florian Rader (Cedars-Sinai Medical Center, USA), Sounok Sen (Yale School of Medicine, USA), Patrick Bering (MedStar Washington Hospital Center, USA), Mathew Maurer (New York-Presbyterian/Columbia University Medical Centre, USA), Sumeet Mitter (Mount Sinai Hospital, USA), Mark Sherrid (NYU Langone Medical Center, USA), Timothy Wong (University of Pittsburg Medical Center, USA), Zainal Hussain (Ascension St. John Clinical Research Institute, USA), Sara Saberi (University of Michigan Health System, USA), Srihari Naidu (Westchester Medical Center, USA), and Jorge Silva Enciso (University of California—San Diego, USA).
