# Supplementary material for: Cardiac biomarkers and effects of aficamten in obstructive hypertrophic cardiomyopathy: the SEQUOIA-HCM trial
Source: Eur Heart J. 2024 Sep 1;45(42):4464–78. doi: 10.1093/eurheartj/ehae590 (PMC11544315; doi:10.1093/eurheartj/ehae590)
Supplement: ehae590_Supplementary_Data [file ehae590_supplementary_data.zip › ehae590_Supplementary_Data.pdf]

Supplementary Appendix

Cardiac biomarkers and effect of aficamten in obstructive hypertrophic cardiomyopathy: the SEQUOIA-HCM trial

Caroline J. Coats[1], Ahmad Masri[2], Roberto Barriales-Villa[3], Theodore P. Abraham[4], D. Marshall Brinkley[5], Brian L. Claggett[6], Albert Hagege[7], Sheila M. Hegde[6], Carolyn Y. Ho[8], Ian J. Kulac[6], Matthew M. Y. Lee[1], Martin S. Maron[9], Iacopo Olivotto[10], Anjali T. Owens[11], Scott D. Solomon[6], Jacob Tfelt-Hansen[12], Hugh C. Watkins[13], Daniel L. Jacoby[14], Stephen B. Heitner[14], Stuart Kupfer[14], Fady I. Malik[14], Lisa Meng[14], Amy Wohltman[14], and James L. Januzzi[15,16]; on behalf of the SEQUOIA-HCM Investigators

[1]School of Cardiovascular and Metabolic Health, University of Glasgow, Glasgow, UK; [2]Oregon Health & Science University, Portland, OR, USA; [3]Complexo Hospitalario Universitario A Coruña, INIBIC, CIBERCV-ISCIII, A Coruña, Spain; [4]University of California San Francisco, San Francisco, CA, USA; [5]Vanderbilt Heart & Vascular Institute, Nashville, TN, USA; [6]Cardiovascular Division, Brigham and Women's Hospital, Harvard Medical School, Boston, MA, USA; [7]Assistance Publique Hôpitaux de Paris, Hôpital Européen Georges-Pompidou, Département de Cardiologie, Paris, France; [8]Department of Medicine, Brigham and Women's Hospital, Harvard Medical School, Boston, MA, USA; [9]Lahey Hospital and Medical Center, Burlington, MA, USA; [10]Meyer Children's Hospital, Istituto di Ricovero e Cura a Carattere Scientifico, Florence, Italy; [11]University of Pennsylvania Perelman School of Medicine, Philadelphia, PA, USA; [12]Section of Forensic Genetics, Department of Forensic Medicine, Faculty of Health and Medical Sciences, University of Copenhagen, and Department of

Cardiology, Copenhagen University Hospital Rigshospitalet, Copenhagen, Denmark; [13]Radcliffe Department of Medicine, University of Oxford, Oxford, UK; [14]Cytokinetics, Incorporated, South San Francisco, CA, USA; [15]Division of Cardiology, Department of Medicine, Massachusetts General Hospital and Harvard Medical School, Boston, MA, USA; and [16]Baim Institute for Clinical Research, Boston, MA, USA

**Supplementary**	**Page**
Figure S1	3
Figure S2	4
Figure S3	5
Figure S4	6
Figure S5	7
Figure S6	8
Figure S7	9
Figure S8	10
Figure S9	11-12
Table S1	13
Table S2	14

Supplementary Figure S1 (A) Study schema. Black arrows indicate timing of clinical and biomarker assessments. (B) CONSORT diagram for the SEQUOIA-HCM biomarker analysis. hs-cTnI, high-sensitivity cardiac troponin I; NT-proBNP, N-terminal pro-B-type natriuretic peptide; SoC, standard of care.

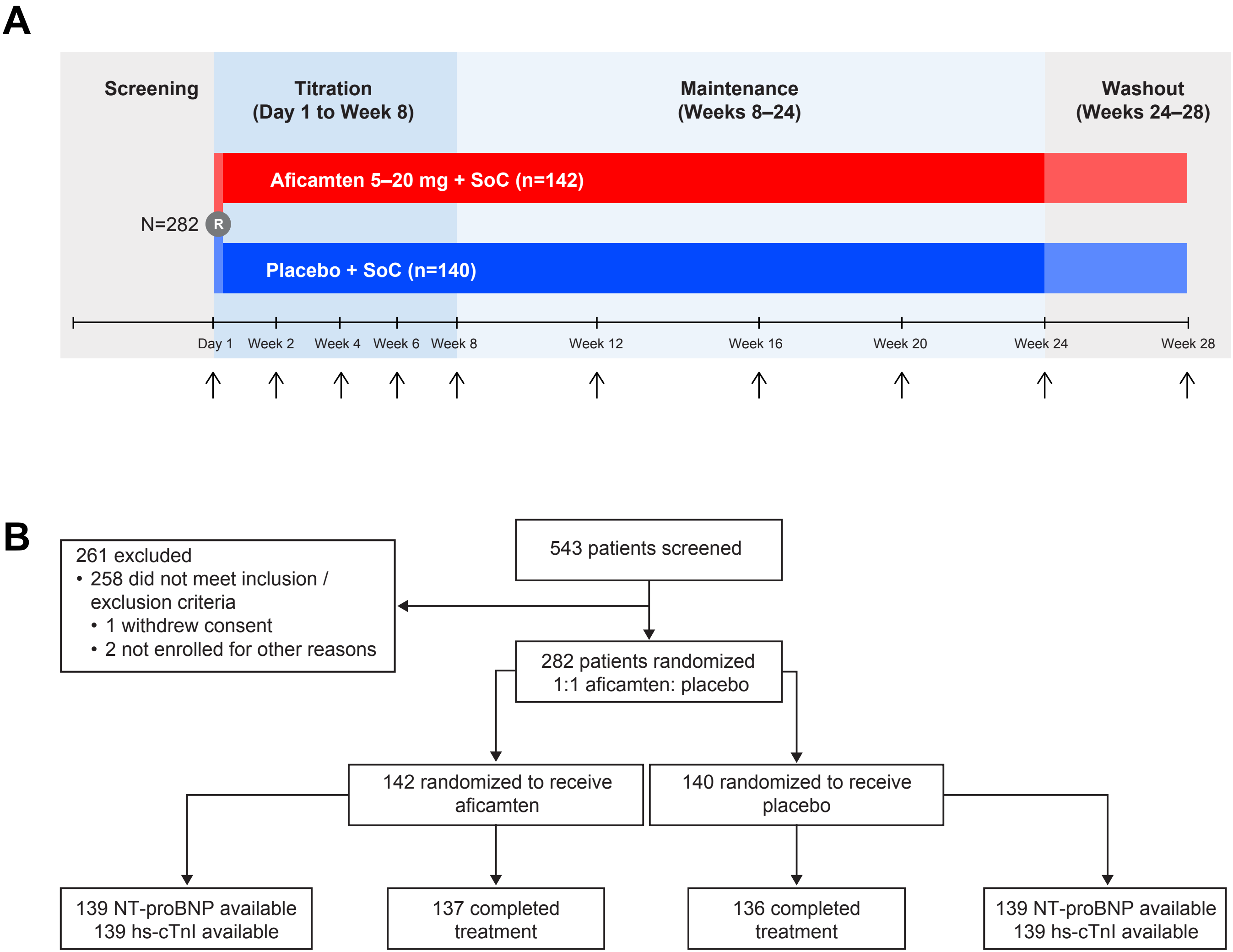

Supplementary Figure S2 Cubic spline graphs detailing univariate associations between baseline concentrations of NT-proBNP and key clinical characteristics of oHCM including (A) maximal wall thickness (B) BSA LAVi (C) LV mass index (D) E/e´ septal (E) BSA LVEDVi (F) LVEF (G) Valsalva LVOT-G (H) and Resting LVOT-G. Histograms show the distribution in NT-proBNP concentration. BSA, body surface area; E/e´, ratio of early diastolic mitral inflow velocity to early diastolic mitral annulus velocity at the septum; LAVi, left atrial volume index; LV, left ventricular; LVEDVi, left ventricular end-diastolic volume index; LVEF, left ventricular ejection fraction; LVOT-G, left ventricular outflow tract gradient; NT-proBNP, N-terminal pro-B-type natriuretic peptide; oHCM, obstructive hypertrophic cardiomyopathy.

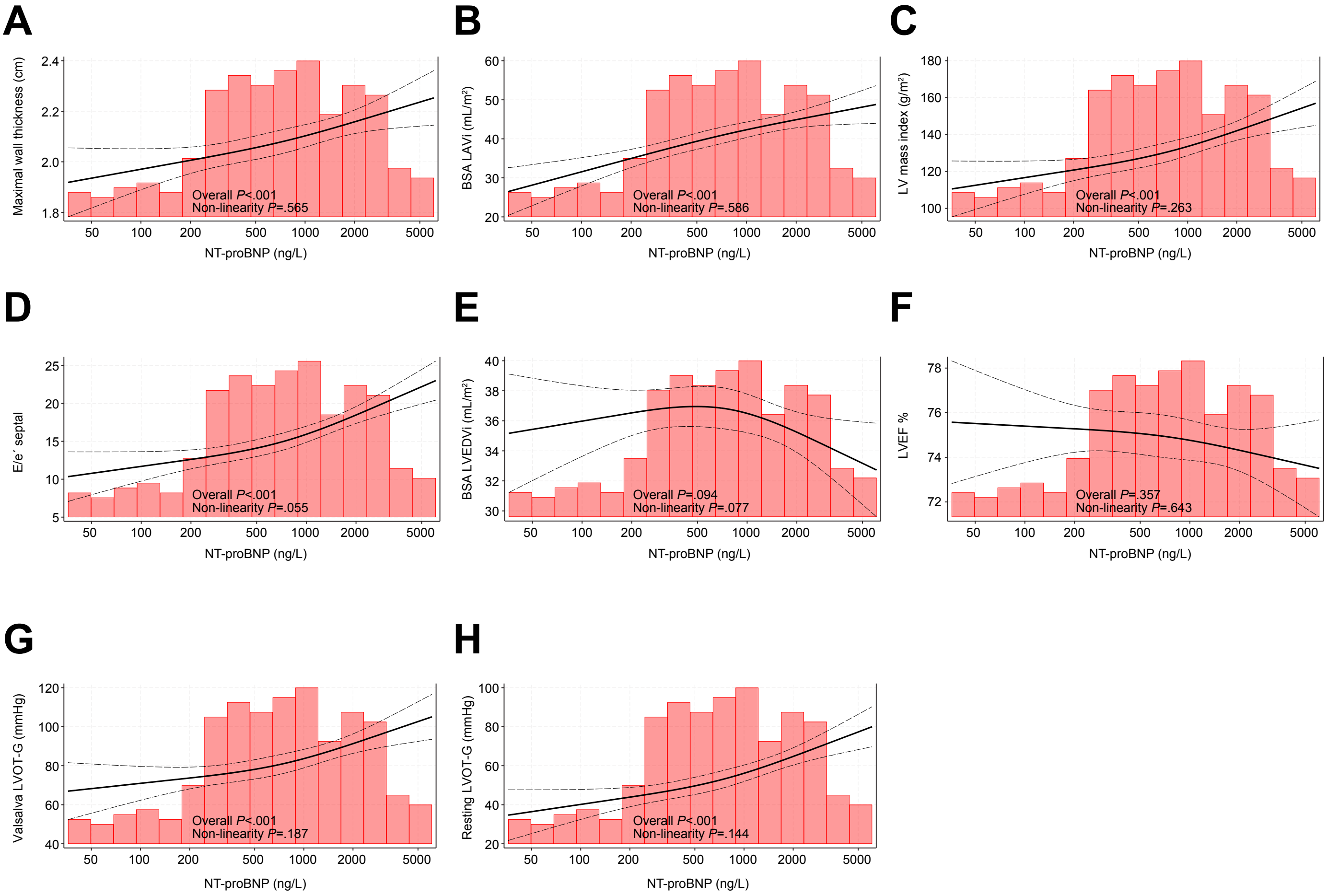

Supplementary Figure S3 Cubic spline graphs detailing univariate associations between baseline concentrations of hs-cTnI and key clinical characteristics of oHCM including (A) maximal wall thickness (B) BSA LAVi (C) LV mass index (D) E/e´ septal (E) BSA LVEDVi (F) LVEF (G) Valsalva LVOT-G (H) Resting LVOT-G. Histograms show the distribution in hs-cTnI concentration. BSA, body surface area; E/e´, ratio of early diastolic mitral inflow velocity to early diastolic mitral annulus velocity at the septum; hs-cTnI, high-sensitivity cardiac troponin I; LAVi, left atrial volume index; LV, left ventricular; LVEDVi, left ventricular end-diastolic volume index; LVEF, left ventricular ejection fraction; LVOT-G, left ventricular outflow tract gradient; oHCM, obstructive hypertrophic cardiomyopathy.

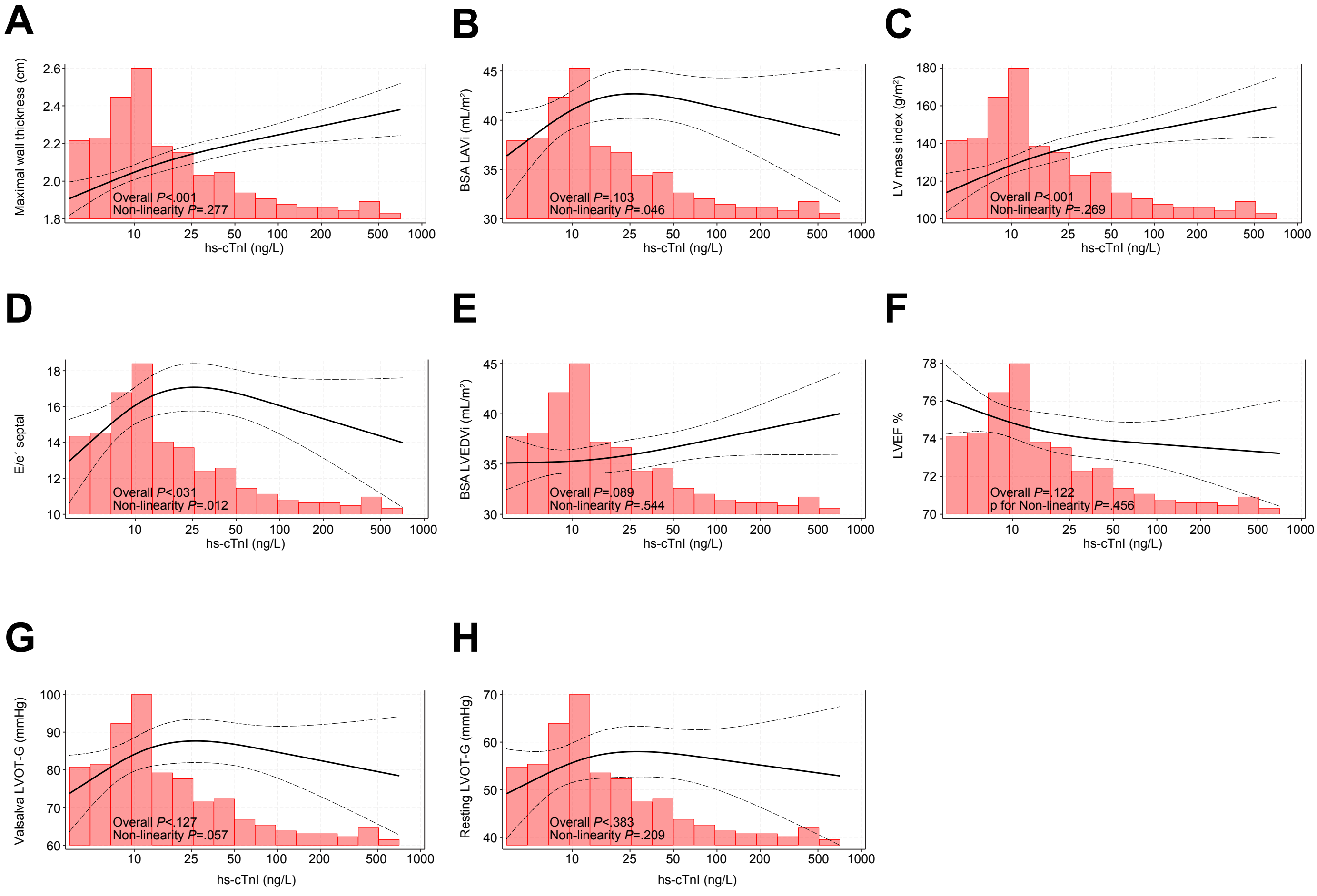

Supplementary Figure S4 Forest plot showing the improvement in exercise capacity is consistent in the biomarker sub-groups. hs-cTnI, high-sensitivity cardiac troponin I; NT-proBNP, N-terminal pro-B-type natriuretic peptide; pVO_2, peak oxygen uptake. Dotted vertical line at 0 indicates no change from baseline.

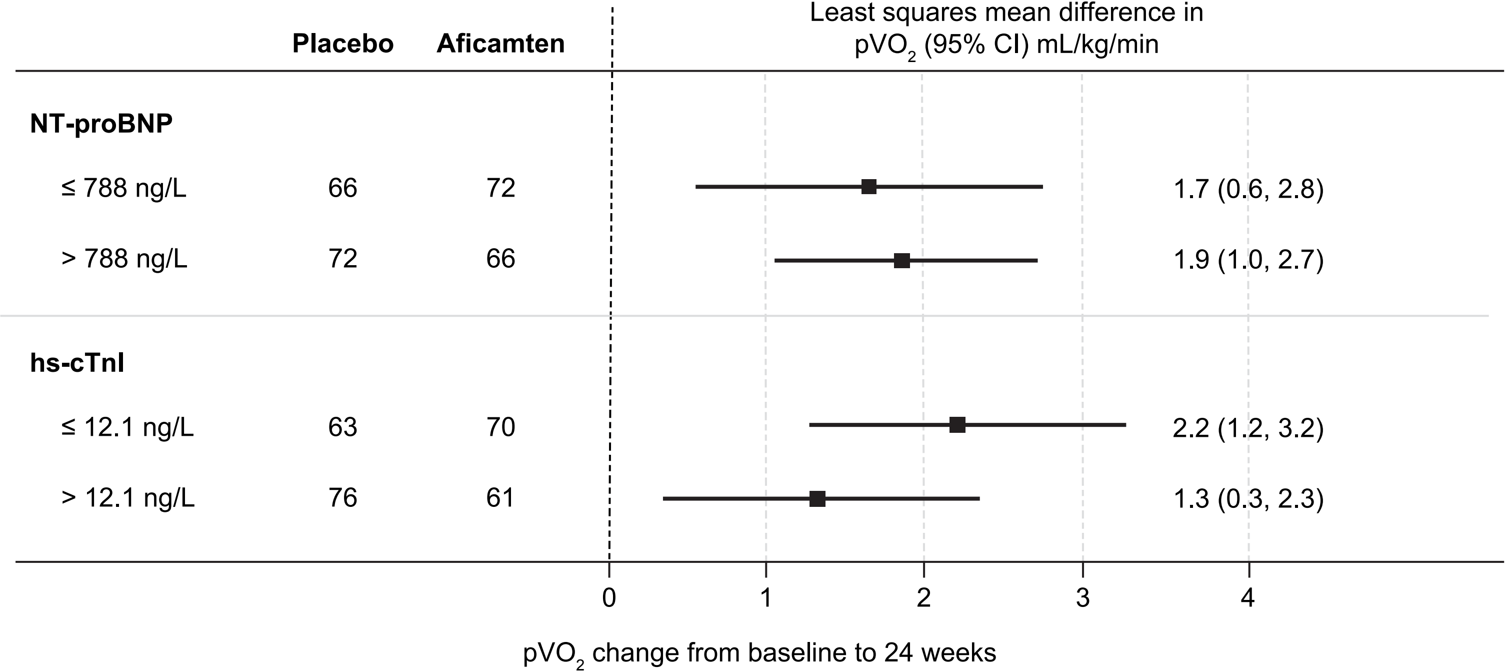

Supplementary Figure S5 Effect of aficamten vs. placebo on the relationship between the change in NT-proBNP and change in Valsalva LVOT-G at 24 weeks. Solid black vertical line indicates no treatment effect. LVOT-G, left ventricular outflow tract gradient; NT-proBNP, N-terminal pro-B-type natriuretic peptide.

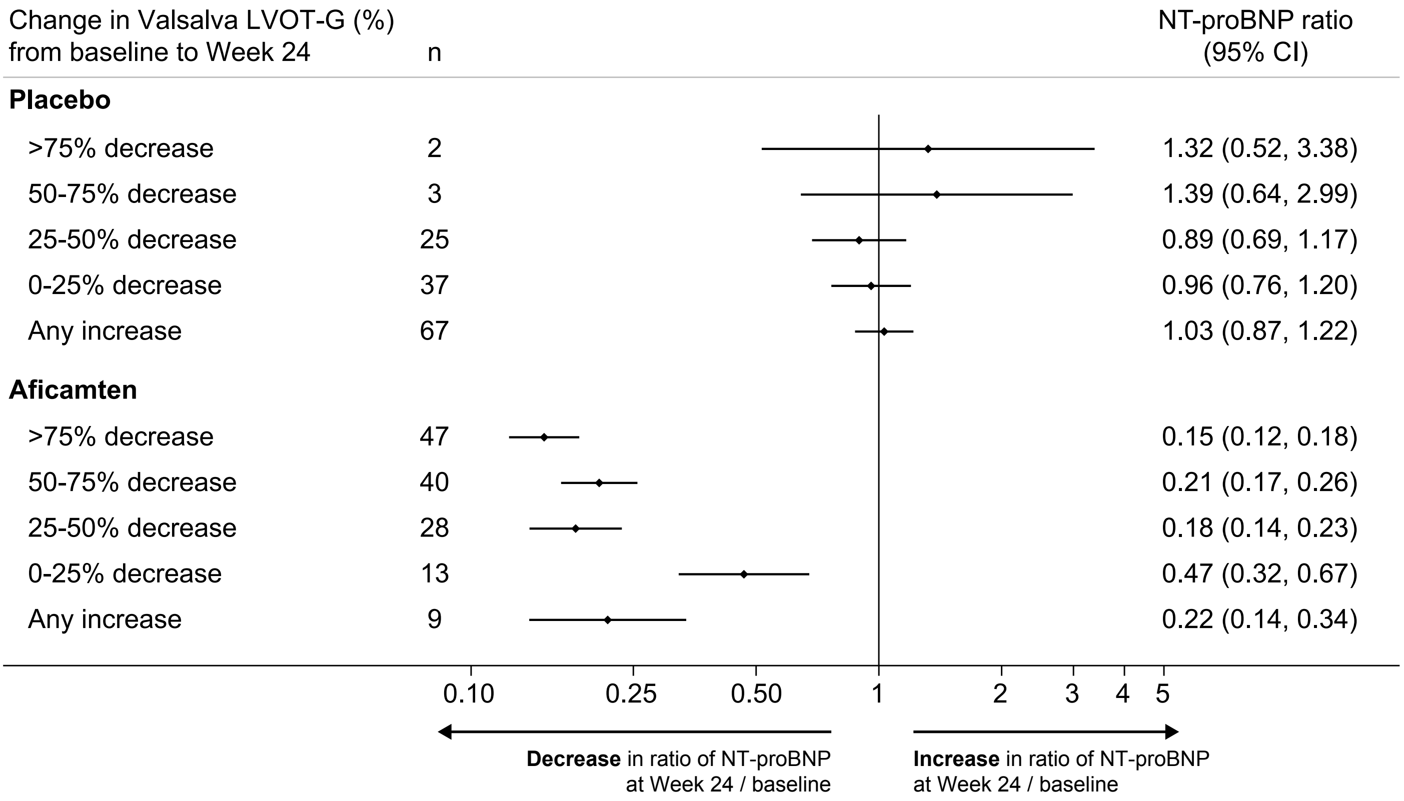

Supplementary Figure S6 Cubic splines analysis relating change in NT-proBNP concentration from baseline to Week 2 with key measures in oHCM, including (A) pVO$_2$ (B) Valsalva LVOT-G (C) maximal LV wall thickness (D) E/e´ septal (E) LAVi and (F) KCCQ-CSS at Week 24. Changes in NT-proBNP as early as 2 weeks after treatment were substantially correlated with changes in key measures in oHCM. Solid and dotted lines show the association correlate with 95% CIs. Black lines indicate no change from baseline. Blue is placebo arm. Red is aficamten arm. BSA, body surface area; CI, confidence interval; E/e´, the ratio of early diastolic mitral inflow velocity to early diastolic mitral annulus velocity at the septum; KCCQ-CSS, Kansas City Cardiomyopathy Questionnaire Clinical Summary Score; LAVi, left atrial volume index; LV, left ventricular; LVOT-G, left ventricular outflow tract gradient; NT-proBNP, N-terminal pro-B-type natriuretic peptide; oHCM, obstructive hypertrophic cardiomyopathy; pVO$_2$, peak oxygen uptake.

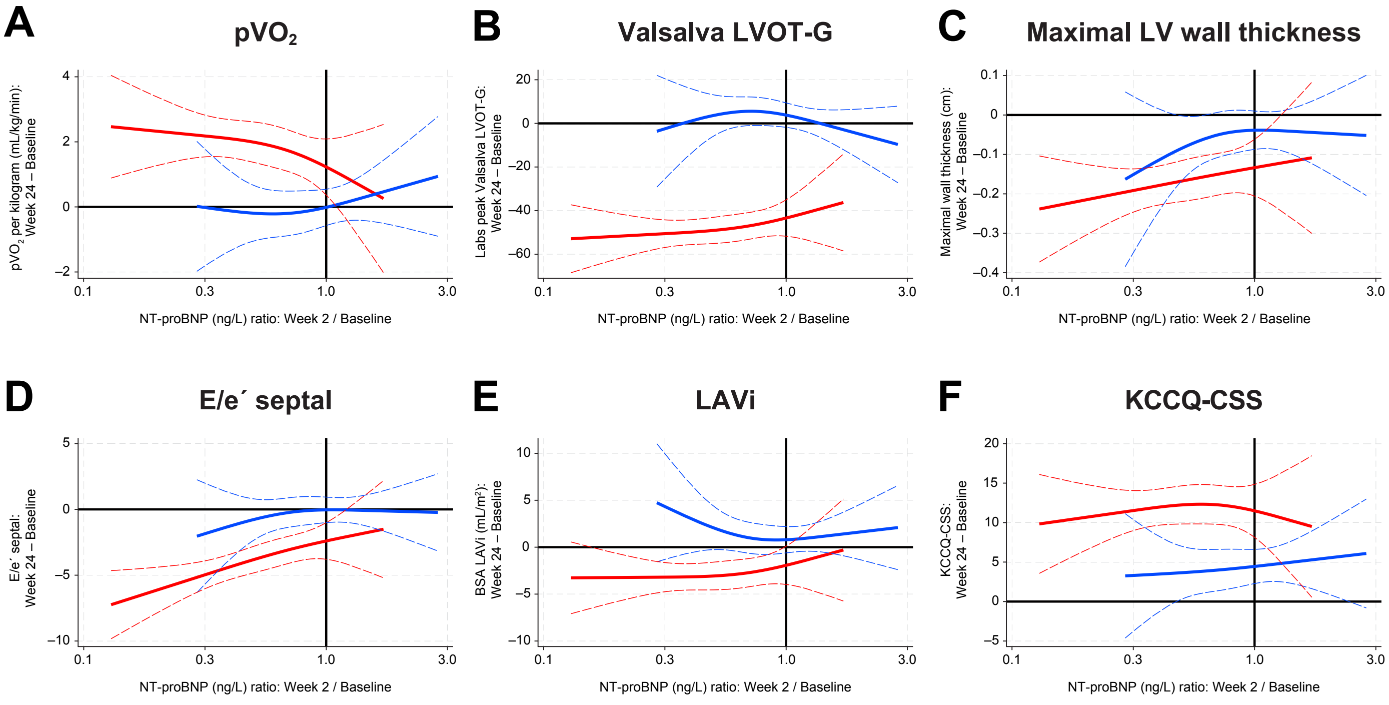

Supplementary Figure S7 Cubic splines analysis relating change in hs-cTnI concentration from baseline to Week 2 with key measures in oHCM, including (A) pVO_2 (B) Valsalva LVOT-G (C) maximal LV wall thickness (D) E/e´ septal (E) LAVi and (F) KCCQ-CSS at Week 24. Changes in hs-cTnI as early as 2 weeks after treatment were substantially correlated with changes in key measures in oHCM. Solid and dotted lines show the association correlate with 95% CIs. Black lines indicate no change from baseline. Blue is placebo arm. Red is aficamten arm. BSA, body surface area; CI, confidence interval; E/e´, the ratio of early diastolic mitral inflow velocity to early diastolic mitral annulus velocity at the septum; KCCQ, Kansas City Cardiomyopathy Questionnaire Clinical Summary Score; LAVi, left atrial volume index; LV, left ventricular; LVOT-G, left ventricular outflow tract gradient; NT-proBNP, N-terminal pro-B-type natriuretic peptide; oHCM, obstructive hypertrophic cardiomyopathy; pVO_2, peak oxygen uptake.

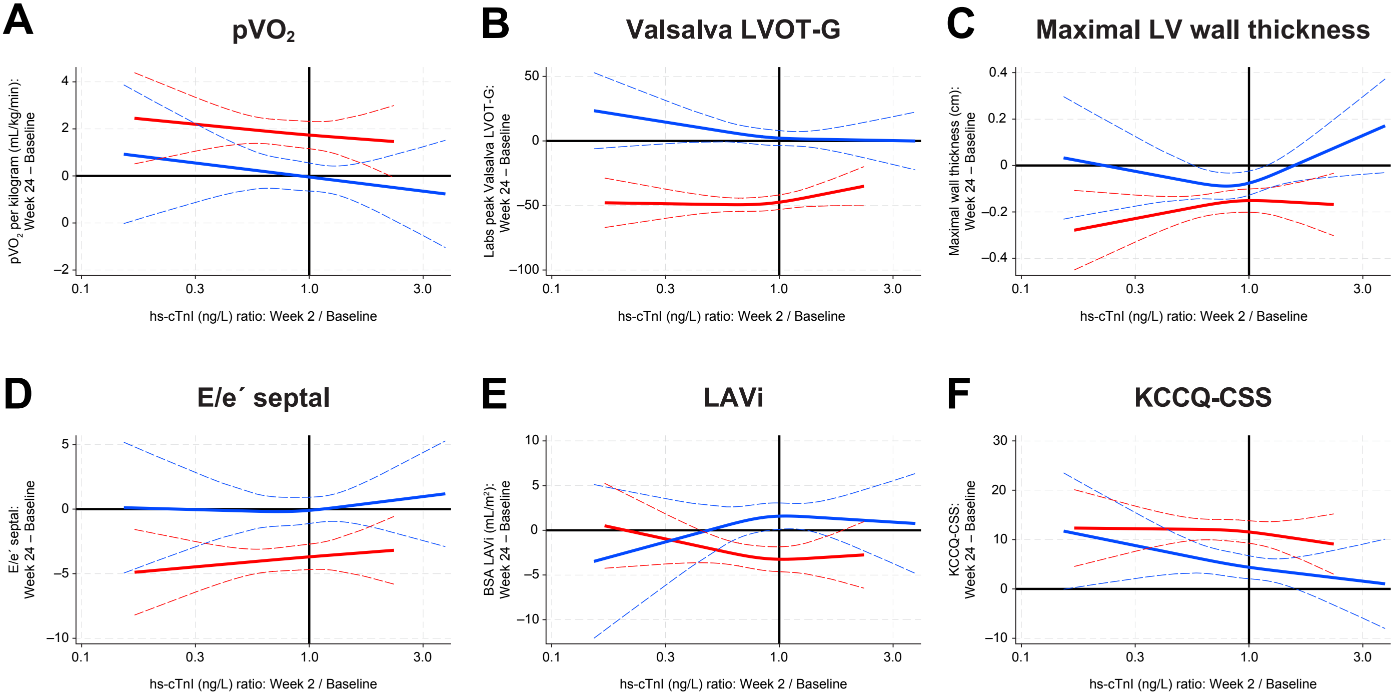

Supplementary Figure S8 Correlation between changes in (A) NT-proBNP and (B) hs-cTnI concentrations and change in LVEF. A direct association was present after treatment initiation. Black lines indicate no change from baseline. Blue is placebo arm. Red is aficamten arm. hs-cTnI, high-sensitivity cardiac troponin I; LVEF left ventricular ejection fraction; NT-proBNP, N-terminal pro-B-type natriuretic peptide.

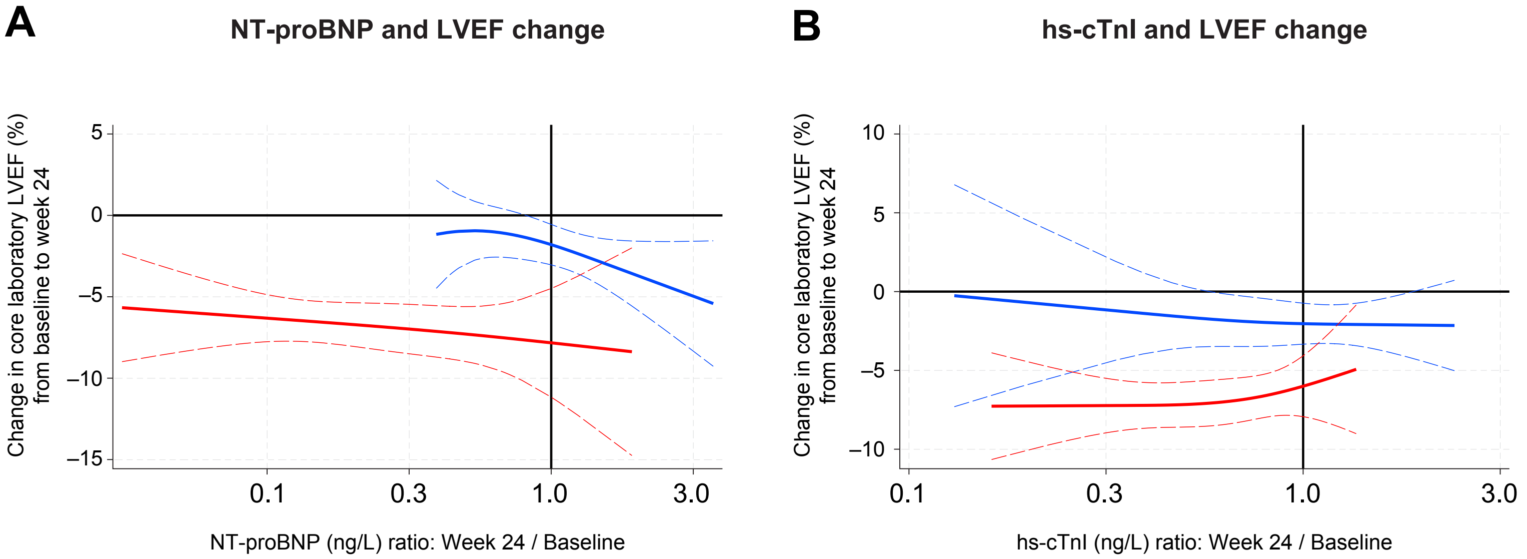

Supplementary Figure S9 Scatterplots of individual patient data at baseline and Week 24 in the (A) aficamten arm and (B) placebo arm. Females are represented by pink dots and males by blue dots. hs-cTnI, high-sensitivity cardiac troponin I; LVEF, left ventricular ejection fraction; LVOT-G, left ventricular outflow tract gradient; NT-proBNP, N-terminal pro-B-type natriuretic peptide; pVO_2, peak oxygen uptake.

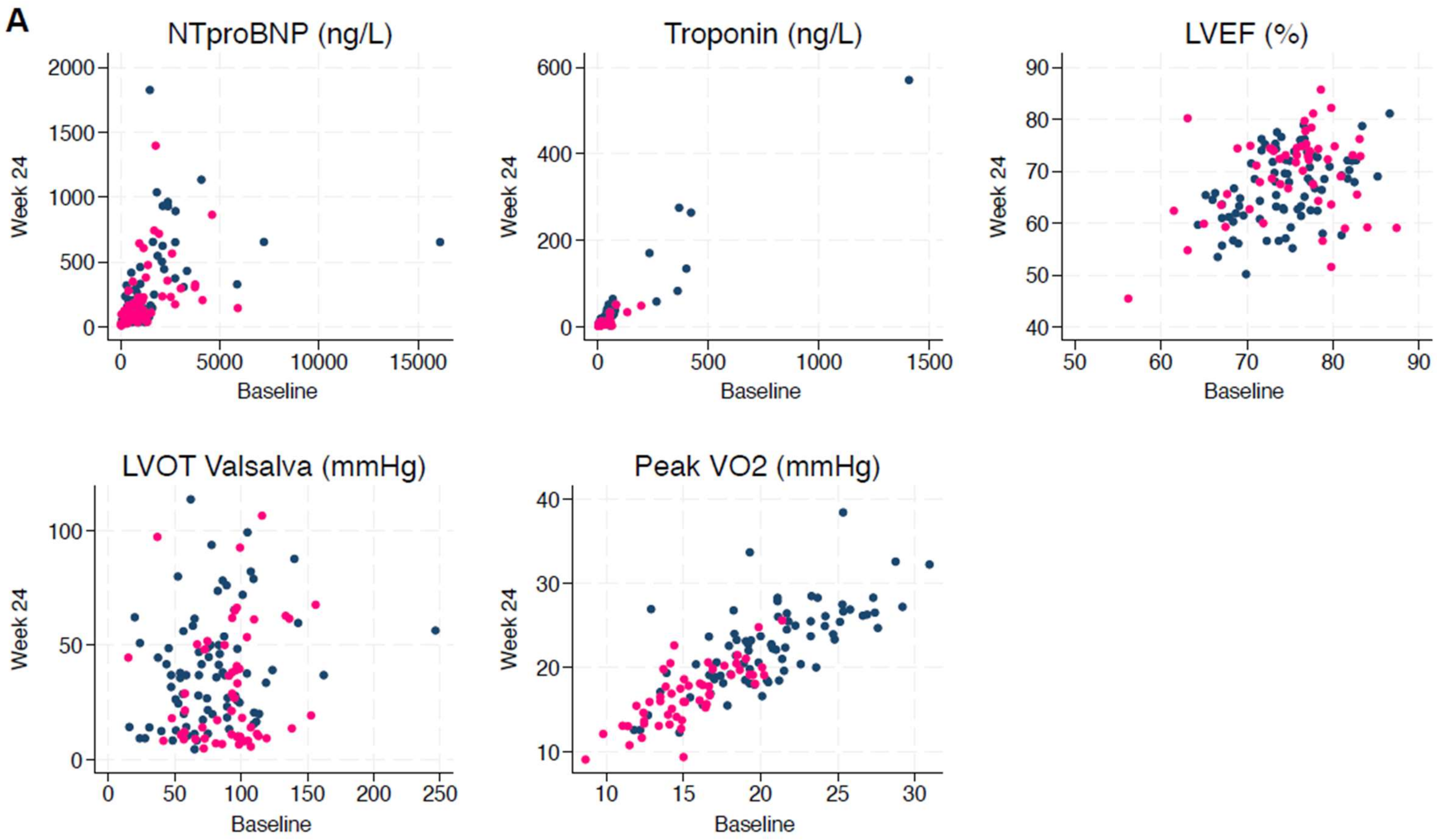

Supplementary Figure S9 Scatterplots of individual patient data at baseline and Week 24 in the (A) aficamten arm and (B) placebo arm. Females are represented by pink dots and males by blue dots. hs-cTnI, high-sensitivity cardiac troponin I; LVEF, left ventricular ejection fraction; LVOT-G, left ventricular outflow tract gradient; NT-proBNP, N-terminal pro-B-type natriuretic peptide; pVO_2, peak oxygen uptake.

B

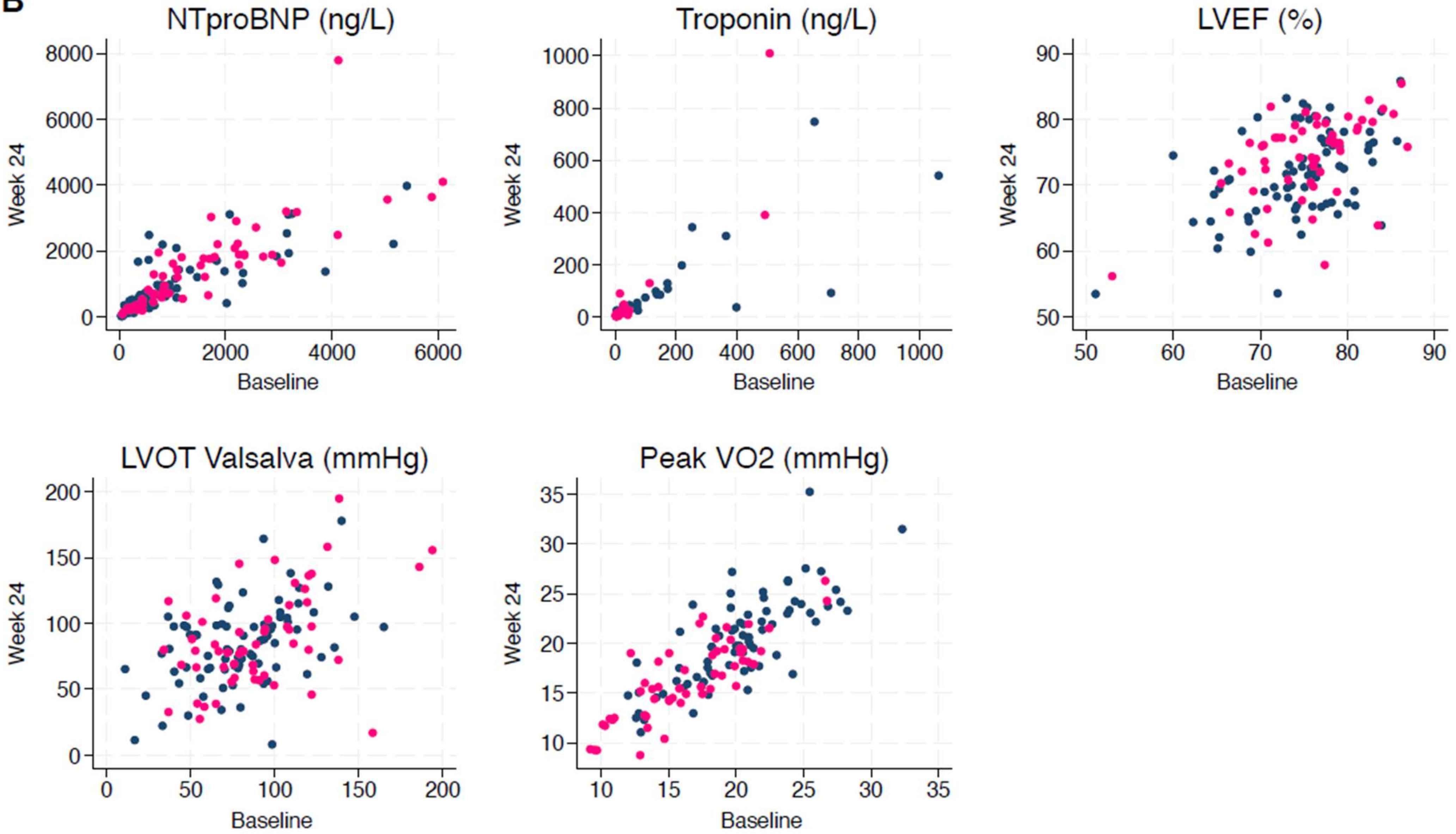

Supplementary Table S1. Predictors of baseline NT-proBNP (excluding hs-TnI)

	Univariate			Multivariable		
**Covariate**	**Association (95% CI)**	**|Z|**	***P*-value**	**Association (95% CI)**	**|Z|**	***P*-value**
E/e´ septal (per SD)	+69% (+50%, +91%)	8.7	<.001	+42% (+26%, +59%)	5.9	<.001
BMI (per 5 kg/m^2)	−36% (−46%, −23%)	5.0	<.001	−30% (−39%, −19%)	4.9	<.001
LAVi (per SD)	+50% (+32%, +70%)	6.3	<.001	+29% (+16%, +44%)	4.6	<.001
Max LV wall thickness (per SD)	+33% (+17%, +52%)	4.3	<.001	+27% (+14%, +41%)	4.4	<.001
LVOT-G at rest (per SD)	+49% (+31%, +70%)	6.2	<.001	+23% (+10%, +38%)	3.5	<.001
Female sex	+58% (+20%, +107%)	3.3	.001			
E/e´ lateral (per SD)	+55% (+37%, +76%)	6.9	<.001			
LVMi (per SD)	+43% (+25%, +62%)	5.4	<.001			
LVOT-G Valsalva (per SD)	+36% (+20%, +55%)	4.7	<.001			
History of AF	+57% (+7%, +129%)	2.3	.021			
LVEF (per SD)	−9% (−20%, +4%)	1.4	.18			
LVEDVi (per SD)	−8% (−20%, +5%)	1.3	.21			
NYHA class	+21% (−11%, +65%)	1.2	.22			
Age (per 10 years)	+5% (−6%, +16%)	0.8	.40			
Creatinine (per SD)	−1% (−13%, +14%)	0.1	.93			

AF, atrial fibrillation; BMI, body mass index; CI, confidence interval; E/e´, ratio of early diastolic mitral inflow velocity to early diastolic mitral annulus velocity; hs-cTnI, high-sensitivity cardiac troponin I; LAVi, left atrial volume index; LV, left ventricular; LVEDVi, left ventricular end-diastolic volume index; LVEF, left ventricular ejection fraction; LVOT-G, left ventricular outflow tract gradient; LVMi, left ventricular mass index; NT-proBNP, N-terminal pro-B-type natriuretic peptide; NYHA, New York Heart Association; SD, standard deviation.

Supplementary Table S2. Predictors of baseline hs-cTnI (excluding NT-proBNP)

	Univariate			Multivariable		
**Covariate**	**Association (95% CI)**	**|Z|**	***P*-value**	**Association (95% CI)**	**|Z|**	***P*-value**
Max LV wall thickness (per SD)	+54% (+35%, +76%)	6.3	<.001	+54% (+35%, +76%)	6.3	<.001
NT-proBNP (per log)	+41% (+25%, +60%)	5.5	<.001			
LVMi (per SD)	+45% (+27%, +67%)	5.3	<.001			
Female sex	−42% (−57%, −22%)	3.7	<.001			
Age (per 10 years)	−18% (−26%, −8%)	3.4	.001			
LVEDVi (per SD)	+17% (+1%, +35%)	2.1	.035			
LVEF (per SD)	−13% (−25%, +0%)	1.9	.06			
BMI (per 5 kg/m^2)	−16% (−31%, +2%)	1.8	.08			
NYHA class	−12% (−37%, +22%)	0.8	.44			
Creatinine (per SD)	+6% (−8%, +22%)	0.8	.45			
LAVi (per SD)	+6% (−9%, +22%)	0.8	.45			
E/e’ lateral (per SD)	+6% (−9%, +23%)	0.7	.46			
LVOT-G Valsalva (per SD)	+5% (−9%, +22%)	0.7	.47			
E/e’ septal (per SD)	+4% (−10%, +21%)	0.6	.56			
LVOT-G at rest (per SD)	+4% (−10%, +21%)	0.6	.56			
History of AF	−8% (−39%, +38%)	0.4	.68			

AF, atrial fibrillation; BMI, body mass index; CI, confidence interval; E/e´, ratio of early diastolic mitral inflow velocity to early diastolic mitral annulus velocity; hs-cTnI, high-sensitivity cardiac troponin I; LAVi, left atrial volume index; LV, left ventricular; LVEDVi, left ventricular end-diastolic volume index; LVEF, left ventricular ejection fraction; LVOT-G, left ventricular outflow tract gradient; LVMi, left ventricular mass index; NT-proBNP, N-terminal pro-B-type natriuretic peptide; NYHA, New York Heart Association; SD, standard deviation.
