# Supplementary material for: Cardiac biomarkers and effects of aficamten in obstructive hypertrophic cardiomyopathy: the SEQUOIA-HCM trial
Source: Eur Heart J. 2024 Sep 1;45(42):4464–78. doi: 10.1093/eurheartj/ehae590 (PMC11544315; doi:10.1093/eurheartj/ehae590)
Supplement: ehae590_Supplementary_Data [file ehae590_supplementary_data.zip › SEQUOIA-HCM Academic SAP_12_15_23_FINAL_Signature_redacted printed.pdf]

DocuSign Envelope ID: 92ACD458-5612-449B-A2CF-97F3C0AA2665

SEQUOIA-HCM: Academic Statistical Analysis Plan

SEQUOIA-HCM PUBLICATION COMMITTEE CHAIR

[redacted]

Contents

[redacted]

2.6 Cardiac Biomarkers 6

[redacted]

3. SUBGROUPS 8

3.1 Subgroups already delineated in the primary SAP include 9

3.2 Additional subgroups to be included in this analysis 9

4. ALTERNATIVE ANALYTIC APPROACHES 10

DocuSign Envelope ID: 92ACD458-5612-449B-A2CF-97F3C0AA2665

1. INTRODUCTION

[redacted]

[redacted]

[redacted]

[redacted]

This academic SAP (aSAP) lists pre-specified analyses developed by the Publication Committee in collaboration with proposed lead authors.

DocuSign Envelope ID: 92ACD458-5612-449B-A2CF-97F3C0AA2665

2. CLINICAL ENDPOINTS OF INTEREST

<u>In addition to the efficacy and safety variables listed in the trial SAP</u>, the effect of aficamten on the following endpoints below will be explored. All variables and comparisons are between aficamten and placebo treated patients from Baseline to Week 24 unless otherwise stated. All endpoints will be assessed in the full cohort using intention to treat analyses where appropriate. These include:

[redacted]

DocuSign Envelope ID: 92ACD458-5612-449B-A2CF-97F3C0AA2665

- [redacted]

[redacted]

[redacted]
    [redacted]

[redacted]
    [redacted]

[redacted]

[redacted]
    [redacted]

[redacted]

[redacted]

[redacted]
    [redacted]

[redacted]

[redacted]

[redacted]

[redacted]

- [redacted]
- [redacted]
    - [redacted]
- [redacted]

[redacted] [redacted]

- [redacted]
    - [redacted]
- [redacted]
    - [redacted]

[redacted]

[redacted]

2.6 Cardiac Biomarkers

- Change in biomarkers
- Intervisit and overall rates of change in biomarkers from Baseline to Week 28
- Landmark analysis of change in biomarkers by study phase (titration, maintenance, and withdrawal) from Baseline to Week 28
- Time to significant difference in biomarkers between aficamten and placebo
- Efficacy of aficamten by baseline biomarker levels (continuous or quartiles)
- Change in biomarkers by hemodynamic and/or clinical response
- Proportion of safety events by relative and absolute change from baseline in cardiac biomarkers in aficamten versus placebo from Baseline to Week 28
- Correlation between change in biomarkers in aficamten versus placebo from Baseline to Week 24 in echocardiographic, hemodynamic, exercise, and symptom parameters

[redacted]

- [redacted]
- [redacted]
- [redacted]
- [redacted]
- [redacted]
- [redacted]
- [redacted]

[redacted]

- [redacted]
- [redacted]

[redacted]

- [redacted]
- [redacted]

[redacted]

- [redacted]
- [redacted]

[redacted]

- [redacted]
- [redacted]

[redacted].

3. SUBGROUPS

Baseline covariates and subgroups defined in the study SAP, in addition to the subgroups listed below, will be explored to further evaluate the sub-analyses listed above. All subgroups will be identified based on randomization or pre-randomization data unless otherwise specified. For each subgroup, we will assess the treatment effect and interaction with treatment for the primary endpoint and each of the secondary endpoints.

DocuSign Envelope ID: 92ACD458-5612-449B-A2CF-97F3C0AA2665

By default, baseline variables shown in Table 1 for the secondary analyses will mirror those presented in the primary SEQUOIA-HCM manuscript as defined in the primary SAP

Prespecified subgroups for the analysis include, but are not limited to:

3.1 Subgroups already delineated in the primary SAP include:

- Sex
- Age group
- Baseline body mass index
- Baseline NYHA Class
- Baseline KCCQ CSS
- Baseline LVEF
- NT-proBNP
- CPET modality
- Baseline pVO_2
- Beta Blocker use
- Baseline resting LVOT
- Sarcomeric gene mutation status (pathogenic or variant of uncertain significance, and non-disease causing or none)

3.2 Additional subgroups to be included in this analysis*:*

- Septal wall thickness
- HCM morphology (collected in CRF)
- Left atrial volume index
- Mitral regurgitant jet:left atrial area ratio (mitral regurgitation severity)
- LV end diastolic volume
- LV end-systolic volume
- Valsalva LVOT gradient
- Left ventricular ejection fraction
- Global longitudinal strain
- Plasma hsTroponin I concentration
- Blood pressure
- Heart rate
- Non-dihydropyridine calcium-channel blocker use

DocuSign Envelope ID: 92ACD458-5612-449B-A2CF-97F3C0AA2665

- Disopyramide use
- Final dose achieved at Week 24 (5, 10, 15, 20 mg and pooled placebo)
- Subjects who experienced LVEF ≤ 50%
- Subjects who experienced LVEF ≤ 40%
- Geographical region (US, China, ROW)

4. ALTERNATIVE ANALYTIC APPROACHES

Unless otherwise specified, alternative approaches may be considered for the primary and secondary endpoints, including, but not limited to linear regression, logistic regression, generalized estimating equation (GEE), Poisson regression, and negative binomial regression models.
